# Impact of Native Environment in Multiheme-Cytochrome Chains of the MtrCAB Complex

**DOI:** 10.1021/acs.jcim.4c02382

**Published:** 2025-04-25

**Authors:** Sasthi C. Mandal, Ronit Sarangi, Atanu Acharya

**Affiliations:** † Department of Chemistry, Syracuse University, Syracuse, New York 13244, United States; ‡ BioInspired Syracuse, Syracuse University, Syracuse, New York 13244, United States

## Abstract

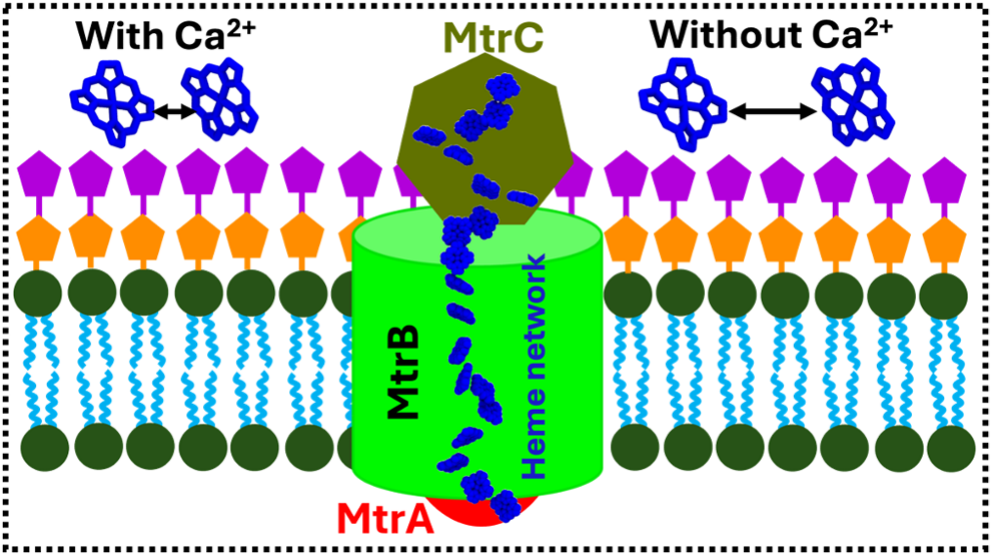

MtrCAB protein complex plays a crucial role in exporting electrons through the outer membrane (OM) to external acceptors. This complex consists of three proteins and contains 20 hemes. Optimal protein–protein interactions and, consequently, heme–heme interactions facilitate efficient electron transfer through the conduit of hemes. The cytochrome MtrA remains mostly inside porin MtrB, and the MtrB barrel contains two calcium ions on its surface. In this study, we investigate the effect of porin-bound calcium ions on the heme–heme distances in the twenty-heme network. We performed all-atom molecular dynamics simulations of the OM–protein complex, MtrCAB, in the presence and absence of the MtrB-bound calcium ions. We observe that the calcium ions bound to MtrB affect the interfacial heme–heme distance when all of the hemes are oxidized and impact one of the heme–heme distances in MtrC when all of the hemes are reduced. In both cases, the absence of calcium ions increases the heme–heme distance, highlighting the crucial role of calcium ions in maintaining the heme network, which is essential for long-range charge transport.

## Introduction

**Figure 1:**
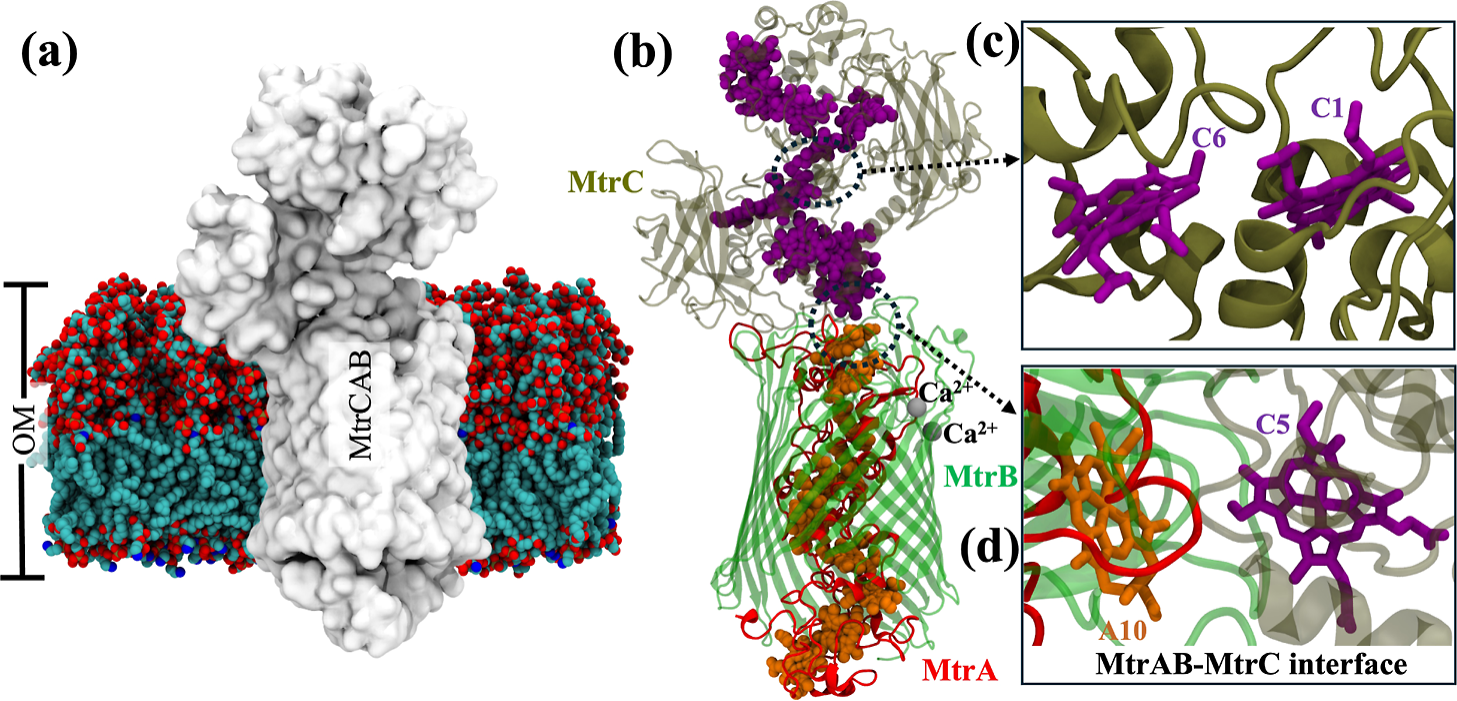
(a) MtrCAB embedded in the OM of *S. oneidensis*. MtrCAB is illustrated in the surface representation. Lipids are shown as spheres. The carbon, nitrogen, oxygen, and phosphorus atoms are illustrated in cyan, blue, red, and tan, respectively. (b) Structure of MtrCAB. MtrA, MtrB, and MtrC are shown in red, green, and tan, respectively. The heme groups of MtrA and MtrC are illustrated in orange and purple, respectively. MtrB-bound calcium ions are shown in silver spheres. Two crucial heme pairs are highlighted: (c) C1–C6 in MtrC and (d) A10-C5 at the MtrA–MtrC interface.

Metal-reducing bacteria can survive in the absence of oxygen by shuttling electrons from inside of the cell to terminal acceptors, such as metal oxides, during respiration.^1,2^ In these Gram-negative bacteria, such as *Shewanella*, electrons cross the outer membrane (OM) to the extracellular substrate through porin cytochrome complexes embedded in the OM.^3^ In *Shewanella oneidensis*, the porin-cytochrome complex, MtrCAB (Figure 1a), is one of the pathways for electrons to be transported across the OM.^3,4^ The MtrCAB complex is made up of a decaheme cytochrome, MtrA, a 26 antiparallel β-strand porin, MtrB, and a second decaheme cytochrome, MtrC, localized on the extracellular domain (Figure 1b,c).^4,5^ MtrA is inserted into the MtrB protein with the N terminus exposed on the periplasm side, while MtrC forms a tight complex with MtrAB, near the C-terminal heme of MtrA.^6^ The distance between the nearest heme groups of MtrA and MtrC is close enough to facilitate electron transfer (ET) through the protein–protein interface (Figure 1b,d).^4^ Since ET rates drop exponentially with increasing heme–heme distance,^7−10^ the distance between the hemes throughout the entire network is crucial to the overall respiration rate of these species.

*Shewanella* transports electrons across micrometer-long distances to external minerals, which act as terminal electron acceptors, using extracellular appendages, called bacterial nanowires.^11−16^ These nanowires act as the extensions of the OM and the periplasm.^17^ Recent computational studies reported that electron transport occurs over long distances through small tetraheme cytochrome (STC) nanowires.^18,19^ Several studies also explored the redox potentials and ET rates of extracellular and periplasmic multiheme cytochromes (MHCs).^4,16,20−24^ However, ET rates at protein–protein interfaces are limited by interprotein heme–heme distances and thermal fluctuations. The impact of interfacial protein–protein, heme–heme, and protein–lipid (Figure 1d) interactions on charge transfer rates in *S. oneidensis* is largely unknown due to the lack of structural information about these complexes. The OM-embedded porin-cytochrome complex MtrCAB has been resolved only recently.^25^

The crystal structure of MtrCAB of *Shewanella baltica* shows two calcium ions attached to the MtrB surface (Figure 1b). A previous study on another β-barrel protein, BtuB, reported the essential role of calcium ions in maintaining the loop conformation for substrate binding.^26^ Therefore, we hypothesize that MtrB-bound calcium ions play a key role in the protein–protein interfacial interactions and, thus, preserve the heme network. The specific role of these MtrB-bound calcium ions in the ET network of MtrCAB has never been studied.

Although significant progress has been made in understanding the ET mechanisms of the MtrCAB complex, most studies have focused on isolated MtrC protein in solution.^27−29^ The influence of OM environments, which likely affect the structure, dynamics, and interactions in the entire complex, has not yet been explored. In this study, we investigate the role of MtrB-bound calcium ions in maintaining the heme–heme distances within MtrA and MtrC and at the MtrA–MtrC interface. Furthermore, we also study the effects of different redox states on this heme network.

## Computational Methods

### System Building

#### Building MtrCAB of *S. oneidensis*

The crystal structure of the MtrCAB complex of *S. oneidensis* is not resolved yet. Therefore, the MtrCAB model of *S. oneidensis* in the OM was built using the recently resolved MtrCAB structure of *S. baltica* (PDB ID: 6R2Q).^25^ Sequence alignment between these two species shows that the MtrA and MtrB sequences^30^ are very similar, while the MtrC sequence is less than 50% similar (Figures S1 and S2). Therefore, we introduced mutations on the *S. baltica* sequence to achieve the *S. oneidensis* model of MtrA and MtrB. Note that the free MtrC structure of *S. oneidensis* is available. The sequence similarity between *S. oneidensis* and *S. baltica* is projected on the structures of MtrA, MtrB, and MtrC separately, as shown in Figure S3. Instead of mutating the*S. baltica* MtrC residues, we aligned the MtrC (PDB ID: 4LM8)^31^ of *S. oneidensis* on the MtrCAB complex of *S. baltica* and prepared the MtrCAB complex model for *S. oneidensis*. The same MtrC structure was also used to model the free MtrC system for simulations. More details of the modeling strategy are provided in Supporting Information.

The MtrA and MtrC proteins contain 10 heme groups each, and each heme is covalently connected to the protein through cysteines and histidines. The charges of the heme and the CYS and HIS residues that are covalently connected to the heme, were taken from ref (20) for both oxidized and reduced states. The parameters for bond, angle, dihedral, and improper taken from ref (20) are the same in the oxidized and reduced states.

#### Building OM–MtrCAB Complex

The OM of *S. oneidensis* was modeled based on the OM of *Escherichia coli* using CHARMM-GUI.^32^ The lipids used in the lower leaflet were PVCL2, PMPE, PMPG, PVPE, and PVPG, with a ratio of 2:8:1:8:2. Since the lipopolysaccharides of *S. oneidensis* MR-1 lack O-antigens,^33^ we constructed the upper leaflet without the O-antigens, akin to the *E. coli* K12 strain. The structures of different lipids used in our study are shown in Figure S4. Finally, to build the OM-MtrCAB complex, the MtrCAB of *S. oneidensis* was inserted into the OM using CHARMM-GUI. Additional details of building the OM-MtrCAB complex are discussed in the Supporting Information. Overall, we built four systems for the MD simulations: OM-MtrCAB with and without MtrB-bound calcium ions, each in oxidized and reduced states. In addition to these four systems, we built free MtrC in an aqueous solution and analyzed it both in the oxidized and reduced states. Note that by “oxidized” we mean that all the hemes in the system are oxidized, and the same holds for “reduced”. The details of the number of different types of lipids in each bilayer, the total number of water molecules, and the total number of atoms for all simulations are shown in Table S1.

### MD Simulations

All simulations were carried out using NAMD^34,35^ with CHARMM36m^36^ and CHARMM36^37^ force field parameters for proteins and lipids, respectively. The TIP3P water model was used in our study.^38^ Langevin thermostat^39^ was used to maintain the temperature at 310 K, while the Langevin piston barostat^40^ was employed to keep the pressure at 1 atm. For nonbonded interactions, a cutoff of 12 Å was applied, incorporating a switching function starting at a distance of 10 Å. Long range electrostatic interactions were calculated using the particle mesh Ewald method.^41^

To equilibrate the system, first, a 10,000-step energy minimization was performed, followed by an equilibrium simulation of 1 ns by restraining the protein with a force constant of 100 kcal/mol Å^2^ and phosphorus atoms of the OM with a force constant of 10 kcal/mol Å^2^. Next, another 10,000-step energy minimization was performed, followed by an equilibrium simulation of 1 ns by releasing everything except protein. Finally, a 1 ns equilibrium simulation was performed by releasing everything. Three independent production runs, each of 1.2 μs, were carried out from the last step of the equilibration for all the systems, with 2 fs time step. We used the last 1.1 μs for each simulation for analysis. A similar protocol was applied for the free MtrC simulation except for constraints on the OM (no OM present in the free MtrC system). In addition to the equilibrium simulations, we performed replica exchange molecular dynamics (REMD)^42,43^ simulations for the oxidized states in the presence of calcium ions.

For REMD simulations, we employed six replicas for each system spanning a temperature range of 300–350 K. A production run of 27 ns was carried out for each replica, resulting in a total REMD simulation time of approximately 162 ns. REMD and equilibrium MD simulation results suggest that the MtrAB–MtrC interfaces are stable and do not significantly impact the protein–protein contact at the interface (Table S2). More details can be found in the Supporting Information (Figures S5–S7).

## Results and Discussion

### Modeled MtrCAB Complex Presents a Stable Heme Network

To ensure the suitability of the protein–protein interfaces and the heme network, we overlay the modeled structure and the hemes from the crystal structure of *S. baltica*. We observed that the interfacial residues (including the loops of MtrB and MtrC) and the hemes have very similar arrangements (Figure S7). We find that the MtrB-bound calcium ions remained stably coordinated throughout the simulations via electrostatic interactions with the residues at the MtrB binding site, closely aligned with their positions in the crystal structure (Figure S8 and Table S3). Most importantly, these calcium-binding residues obtained from the crystal structure and MD simulations are mostly conserved across various species of *Shewanella* (Figure S9). We analyzed the conformations from the REMD and MD simulations to investigate the interface further. We observed that the number of residues (Table S2) in the MtrA–MtrB and MtrB–MtrC interfaces (within 5 Å from each other) is very similar between the simulated (equilibrium MD and REMD), modeled *S. oneidensis*, and *S. baltica* crystal structure of the MtrCAB complex.

**Figure 2:**
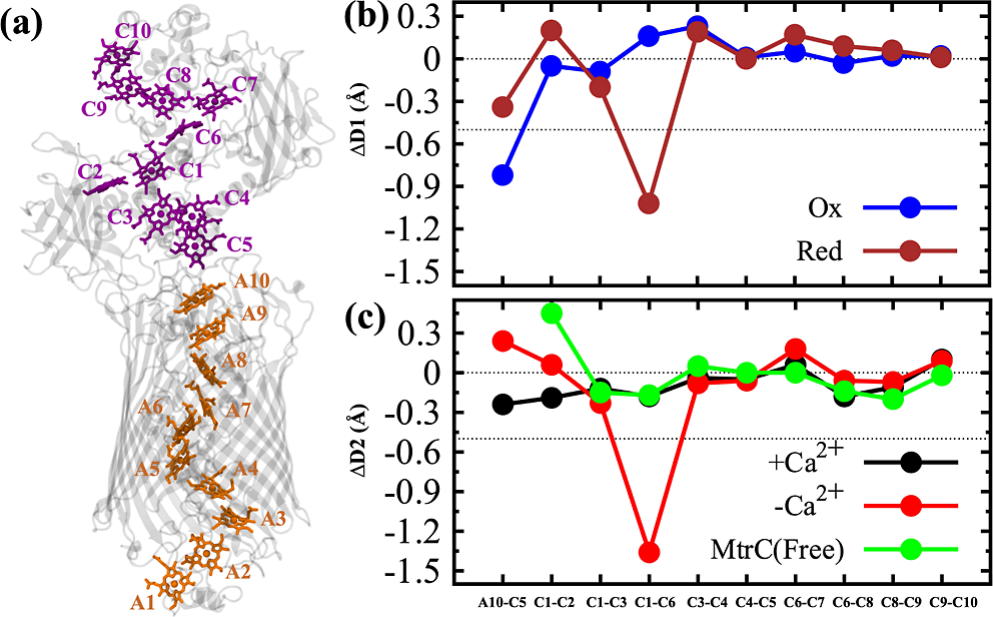
(a) Heme arrangement in the MtrCAB complex. The heme groups of MtrA are shown in orange, while the heme groups of MtrC are shown in purple. (b) Heme–heme distance difference between the presence and absence of calcium ions (Δ*D*1) for the oxidized (blue) and the reduced (brown) states. (c) Heme–heme distance difference between the oxidized and reduced states (Δ*D*2) in the presence (black) and absence (red) of calcium ions. Δ*D*2 for free MtrC are shown in green.

Maintaining heme–heme distances in the MHC (Figure 2a) is necessary as the ET rate decreases exponentially with increasing heme–heme distance. We calculated the edge-to-edge distance between porphyrin rings of two heme groups within individual proteins (MtrA and MtrC) and their interfaces in the presence and absence of MtrB-bound calcium ions and for both oxidation states. The distances were averaged over 3.3 μs simulation trajectories and are shown in a schematic diagram in Figure S10. Since we only want to focus on the changes in heme–heme distances, we analyzed the differences in the same heme–heme distances under different simulation conditions. Similar to the crystal structure, the absolute value of the heme–heme distance is highest for C1–C6 in MtrC.^31^ This larger distance will lead to a lower electronic coupling value for ET between C1 and C6 heme, as reported in a previous study.^24^ Any further increase in this central MtrC heme–heme distance will be detrimental to overall ET.

It is also worth noting that the heme–heme distance at the MtrA–MtrC interface is longer than all other heme–heme distances in MtrCAB, creating a weak spot for the ET chain. A similar observation has also been reported in other MHCs, such as the small tetraheme cytochrome STC.^19^ The average distances between heme pairs based on their arrangements within MtrA and MtrC and between MtrA and MtrC are shown in Table S4. Our analysis suggests that the average distances of the heme pairs are shortest for stacked heme pairs, followed by T-shaped and coplanar pairs, consistent with the trend observed in the crystal structure of MtrCAB. A similar conclusion was reported from the highest electronic coupling for stacked heme pairs, followed by T-shaped and coplanar pairs.^44^ Overall, the simulations do not change the orientations and distance distributions compared to the reported trend observed in either the same or similar proteins.

### Impact of Calcium Ions on the Heme Network in Two Redox States

Since we are interested in the changes in heme–heme distances, a distance difference metric provides more valuable insights than the absolute values of the distances. First, we define a distance difference term Δ*D*1 = Dist (+Ca) – Dist (−Ca) for the same heme pair. Here, +Ca and −Ca refer to simulations in the presence and absence of calcium ions, respectively. A positive value of Δ*D*1 indicates that the heme–heme distance is higher in the presence of calcium ions. In contrast, a negative value indicates that heme–heme distances are higher without calcium ions.

The plot of Δ*D*1 versus heme pairs (A1–A2, A2–A3, ..., A8–A9) is depicted in Figure S11, while the corresponding data for the heme pairs A9–A10 and C1–C2, C2–C3, ..., C9–C10 is shown in Figure 2b. To quantify the impact of these values in the rate constant of ET, we used the Moser–Dutton ruler^45^ (see Supporting Information), and estimated the change in rate as a ratio of rate constants (k_et_^+Ca^/k_et_^–Ca^). In Figure 2b, we marked a dotted line representing Δ*D*1 value of −0.5 Å, which corresponds to k_et_^+Ca^/k_et_^–Ca^ ∼ 2. This indicates that the ET rate for a specific heme pair is twice as high in the presence of calcium ions as the rate in the absence of calcium ions. In the following, we discuss only the heme pairs where |Δ*D*1| ≥ 0.5 Å, corresponding to a k_et_^+Ca^/k_et_^–Ca^ value of 2 or higher.

We observe that the C1–C6, A10–C5, and A1–A2 distances show larger Δ*D*1 values, indicating longer heme–heme distances without calcium ions. The heme pair A1–A2 belongs to the periplasmic region and is exposed to water. Therefore, larger fluctuations are expected for this region and may be necessary to intake electrons from the periplasmic MHCs.^5,46,47^ Remarkably, the changes in the A10–C5 and C1–C6 distances depend on the oxidation state. In the oxidized state, the Δ*D*1 value for A10–C5 is −0.82 Å. Hence, according to the Moser–Dutton ruler, the A10–C5 ET rate decreases by more than 3-fold in the absence of calcium ions. This suggests that MtrB-bound calcium ions are important for ET because they influence the heme–heme spacing at the MtrAB–MtrC interface.

**Figure 3:**
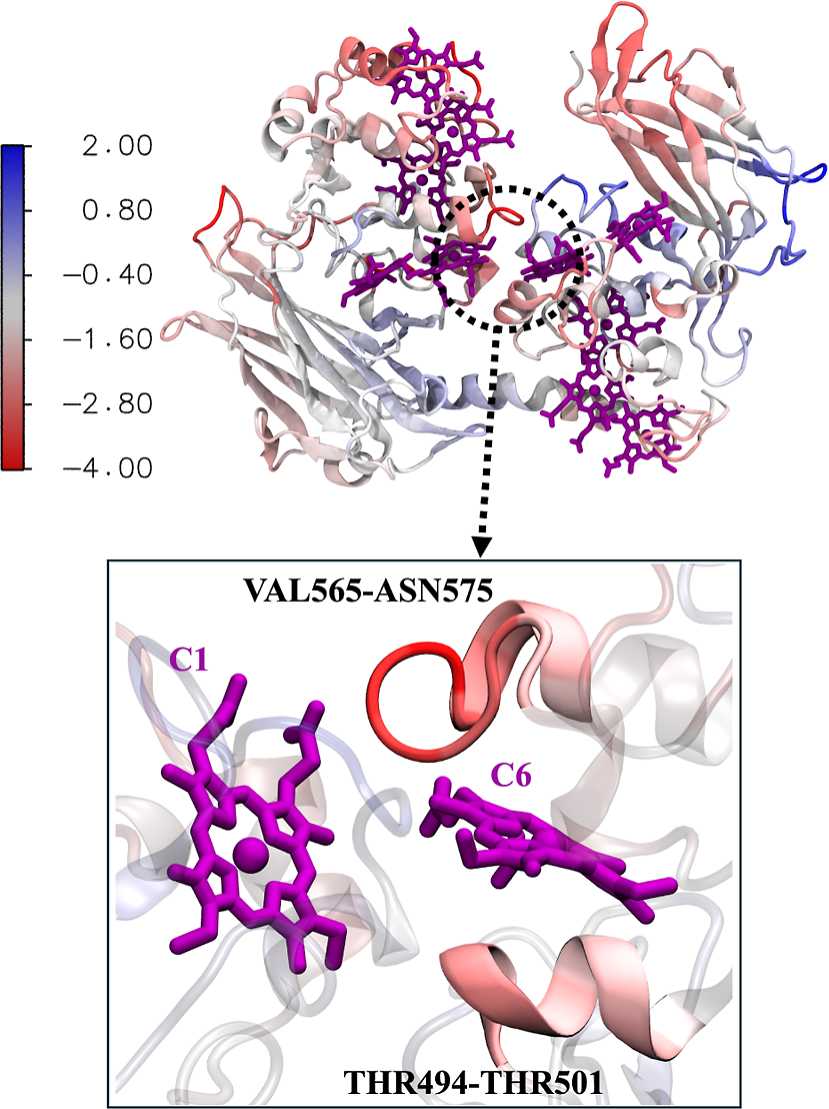
ΔRMSF (= RMSF (+Ca) – RMSF (−Ca)) in the reduced state projected on MtrC. The residues exhibiting higher ΔRMSF around C6 are shown in the zoomed-in view.

The Δ*D*1 values for the heme pairs in MtrC do not show any significant changes in any of the oxidation states except for the C1–C6 pair in the reduced state, which increases by 1.02 Å in the absence of the calcium ions. This indicates that, in the reduced state, the C1–C6 ET rate decreases by more than 4-fold in the absence of calcium ions. We observed that the larger C1–C6 distances without calcium ions originate from larger fluctuations of protein residues around heme C6 (Figure 3). Our RMSF data show that the residues THR494-THR501 and VAL565-ASN575 adjacent to the C6 heme exhibit ΔRMSF in the range −2 to −3 Å and −2 to −4 Å, respectively (Table S5, Figures S12 and S13).

It was previously reported that the efficiency of electron flow is comparable between the perpendicular direction, from C5 to C10 (Figure 2a), and the parallel direction, from C2 to C7 (Figure 2a) relative to the OM.^24^ The direction of the electron flow may also depend on the surface on which the protein is located.^48^ Since the C1–C6 heme pair is central for ET in both directions, a longer C1–C6 distance without calcium will slow down ET irrespective of the direction.

Previous computational study also proposed that the electrons are likely transported from C2 heme to a flavin molecule that binds near the C2 heme.^49^ Note that these previous studies were based on a free MtrC model due to a lack of structural information on the complex. Therefore, other terminal hemes may also be involved in the ET to flavin. For example, soluble substrates may also bind at the heme 7 of MtrF^23^ and UndA.^50^ Overall, our results suggest that the efficiency of electron flow in MtrC is affected along both the parallel and perpendicular directions relative to the OM and can be influenced by the MtrB-bound calcium ions.

### Influence of Oxidation States on the Heme Network

To gain a more comprehensive understanding of the impact of oxidation states on heme–heme distances, we define another distance difference metric, Δ*D*2 = Dist (Ox) − Dist (Red). This quantity can be calculated for MtrCAB simulations with and without calcium ions. In addition, one can also calculate this metric for free MtrC simulations. A positive Δ*D*2 value indicates an increased heme–heme distance in the oxidized state, while a negative value indicates an increase in the reduced state (Figure 2c). Δ*D*2 values for the interfacial heme pair (A10–C5), as well as the heme pairs in MtrC, are generally small, with the notable exception of heme pairs C1–C6, which show a Δ*D*2 value of −1.36 Å. As a result, in the absence of calcium ions, the ET rate decreases by more than 6-fold in the reduced state. However, such a dramatic effect is observed only when the calcium ions are absent. In the presence of the calcium ions, the C1–C6 distance does not change based on the oxidation state. In fact, heme–heme distances obtained from a free MtrC system show a similar Δ*D*2 value with MtrCAB simulations. More detailed analysis of the free MtrC simulations and comparison with MtrCAB simulations are shown in Figures S14–S17. Overall, the oxidation state and the MtrB-bound calcium ions influence the heme–heme distances in the MtrCAB network. Additionally, the results of free MtrC simulations show that the presence of OM and the other proteins also affects heme–heme distances (C1–C6) in the extracellular domain.

### Hydrogen Bonds Play an Important Role in Maintaining the Heme Network

**Figure 4:**
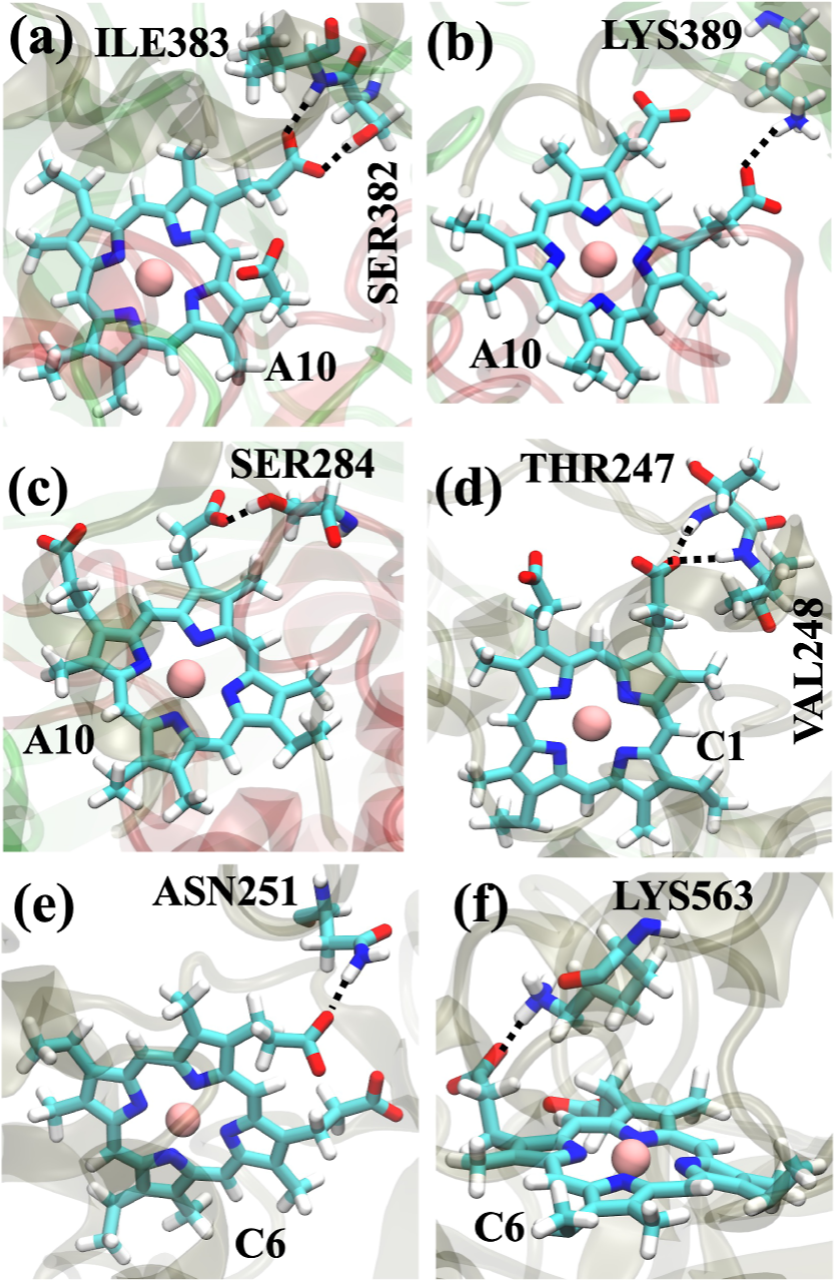
(a,b) Hydrogen bonds between A10 and the residues in MtrB, while the hydrogen bond between A10 and the residue of MtrA is shown in (c) for the oxidized state. In the reduced state, the hydrogen bonds between C1 and MtrC are shown in (d), while those between C6 and MtrC are shown in (e,f).

Hydrogen bonds play an important role in protein–protein and protein–ligand interactions. Although heme propionate side chains have traditionally been seen as mere anchors, they also play a crucial role in maintaining the reactive state of P450cam^51−55^ and heme network in OmcS.^56^ Therefore, we analyzed the hydrogen bonds (Figure 4 and Table S6) formed with the impacted hemes (A10, C5, C1, and C6) from our investigations.

In the oxidized state, the side chain of SER382_MtrB_ and the main chain of ILE383_MtrB_ form transient (with occupancies of 36% and 31%) hydrogen bonds with A10 heme only in the presence of calcium ions (Figure 4a). The hydrogen bonds formed between C1 heme and MtrCAB exhibit similar occupancies in the presence and absence of calcium ions for the oxidized state. The C6 heme forms hydrogen bonds with the side chains of ASN251_MtrC_ (occupancy: 54%) and LYS563_MtrC_ (occupancy: 24%) in the presence of calcium ions, with similar populations even without the calcium ions. The results suggest that in the oxidized state, the hydrogen bond network between the A10 heme and MtrCAB is weaker in the absence of calcium ions compared to the presence of calcium ions. However, the C5 propionates do not form any hydrogen bonds with the protein residues since they are exposed to water. Overall, the lack of hydrogen bonds for A10 heme manifests in an increase in the A10–C5 distances for oxidized MtrCAB without calcium ions. However, since the hydrogen bonds with C1 and C6 hemes are not impacted in the oxidized state by calcium ions, the C1–C6 distance does not change in this oxidation state of the hemes. Removing all side chains in the heme group was shown to significantly slow down the ET rate in MtrCAB,^44^ consistent with our finding.

Now, we discuss the hydrogen bonding interactions between those hemes and MtrCAB in the reduced state. The SER382_MtrB_ and ILE383_MtrB_ form hydrogen bonds with the A10 heme in the presence of calcium, similar to the oxidized case. Interestingly, the A10–SER284_MtrA_ (Figure 4c) and between A10–SER477_MtrB_ hydrogen bonds are not formed in the presence of calcium ions, while they form weak hydrogen bonds in the absence of calcium ions. Therefore, the increase in the A10–C5 distance upon the removal of calcium ions is less pronounced in the reduced state.

The C1 heme of the C1–C6 heme pair forms transient hydrogen bonds in the reduced state with VAL248_MtrC_ and THR247_MtrC_ (Figure 4d), but their population decreases significantly in the absence of the calcium ions (Table S6). In addition, the C6 heme in the reduced state also forms stronger hydrogen bonds with ASN251_MtrC_ (Figure 4e) in the presence of the calcium ions. However, the hydrogen bond between LYS563_MtrC_ and C6 (Figure 4f) shows an opposite trend, with increased occupancy in the absence of calcium ions (Table S6). Overall, C1 and C6 hemes in the reduced state form stronger hydrogen bonds when the calcium ions are present. Consequently, we observed a much longer C1–C6 distance in the reduced state without calcium ions. However, the difference in the number and overall population of the hydrogen bonds are not significantly different between simulations with and without calcium ions in the oxidized state. Therefore, the C1–C6 distances for the oxidized state remain similar in both cases.

### Protein–Protein Interaction Is Stronger in the Presence of Calcium

Protein–protein interactions at the interfaces are crucial for optimizing the heme network in the MtrCAB complex. A weaker interaction between these proteins leads to an increase in the heme–heme distance at the interface, which slows down ET. To investigate this further, we calculated nonbonded interaction energies (van der Waals + electrostatic) between residues in close contact with interfacial hemes. The nonbonded interaction energy data are presented in Table S7. In the oxidized state, the total nonbonded interaction energy in the presence of calcium ions is more than twice as high as in their absence (−48.51 ± 5.3 kcal/mol vs −22.78 ± 8.33 kcal/mol). In contrast, in the reduced state, the difference in total nonbonded interaction energies between the calcium-bound and calcium-free conditions is minimal. The majority of the nonbonded energy contribution for all cases arises from the electrostatic interactions. Therefore, the protein–protein interaction between MtrAB and MtrC is stronger in the presence of calcium ions when all of the hemes are oxidized.

## Summary and Conclusions

In this article, we investigated the role of porin-bound calcium ions in maintaining the optimal heme–heme distances within the MHC complex MtrCAB of *S. oneidensis*. We showed that the heme–heme distance at the interface between two cytochromes is influenced by the calcium ions when all heme groups are oxidized. Therefore, the increase of A10–C5 heme distance in the absence of calcium will exponentially slow down the ET rate in this protein interface. The presence of calcium ions is also important when all the hemes are reduced, since we observe an increase in the central MtrC heme–heme distance without calcium. We have also shown that the residues around the C6 heme of MtrC fluctuate more in the reduced state without the calcium ions, leading to a dramatic increase in the C1–C6 distance in the reduced state. Therefore, the impact of calcium ion on the heme network depends on the oxidation state of the hemes. The oxidized A10 and reduced C1 hemes form stronger hydrogen bonds with protein residues in the presence of the calcium ions. In contrast, the nonbonded interaction at the interface between MtrAB and MtrC is weaker in the oxidized state when calcium ions are absent. Therefore, the lack of hydrogen bonds with these crucial hemes and weaker protein–protein interactions also contribute to the observed increase in the interfacial heme–heme distances based on the oxidation state.

ET between two hemes of the network will impact the overall ET through the micrometer-long cellular appendages involving these MHCs. The direction of the overall charge flow in the overall network is an area of active research.^16,57^ Our data indicates that the long-range electron transport will be hindered significantly when the MtrB-bound calcium ions are absent.

## Data Availability

Models used in this study, including PSF, PDB, and force field parameters, along with initial geometry and final structures of equilibrium and independent production simulations, are available at 10.5281/zenodo.14553204.

## Supporting Information Available

Additional details of modeling, computational details and analysis, sequence alignments, and free MtrC simulations (PDF)
