# Supplementary material for: Impact of Native Environment in Multiheme-Cytochrome Chains of the MtrCAB Complex
Source: J Chem Inf Model. 2025 Apr 25;65(9):4568–75. doi: 10.1021/acs.jcim.4c02382 (PMC12076491; doi:10.1021/acs.jcim.4c02382)
Supplement: Supplementary file 1 — ci4c02382_si_001.pdf [file ci4c02382_si_001.pdf]

Supporting Information: Impact of native environment in multiheme-cytochrome chains of the MtrCAB complex

Sasthi C. Mandal,[†] Ronit Sarangi,[†] and Atanu Acharya[*,†,‡]

†*Department of Chemistry, Syracuse University, Syracuse, NY 13244, United States*

‡*BioInspired Syracuse, Syracuse University, Syracuse, NY 13244, United States*

E-mail: achary01@syr.edu

Modeling MtrCAB and OM-MtrCAB

The structure of the MtrCAB complex of *S. oneidensis* is not available; however, the structure of MtrC has been identified (PDB ID: 4LM8[S1]). Since the sequences of MtrA and MtrB from *S. oneidensis* share similarity with those from *S. baltica*, the MtrAB components of *S. oneidensis* were modeled based on the MtrAB structure from the MtrCAB complex of *S. baltica*. The following steps were used to build the MtrCAB of *S. oneidensis*: First, we added missing residues in MtrAB of *S. baltica*. The residues in the range 1-61 were missing in the MtrA structure of *S. baltica*. Within this region, the segment comprising residues 36–61, predicted with a confidence level exceeding 50 % by the AlphaFold 2[S2] model, was integrated into the crystal structure of MtrA. Subsequently, we performed sequence alignment of MtrA of *S. baltica* with respect to the amino acids sequence of MtrA of *S. oneidensis* using VMD. Based on this alignment, missing residues (74–77 and 113–115) were added, and six residues were mutated in MtrA of *S. baltica* to construct the MtrA of *S. oneidensis*. The amino acid sequences of MtrA for the two species are shown in Figure S1. A similar approach was employed for MtrB, with the amino acid modifications determined based on the sequence alignment of MtrB between *S. oneidensis* and *S. baltica*. Two residues were added, and 77 residues were mutated in the structure of MtrB of *S. baltica* to match the amino acid sequence of MtrB of *S. oneidensis*. The refined structures of MtrA and MtrB were combined using psfgen to generate the MtrAB complex of *S. oneidensis*. The crystal structure of MtrC of *S. oneidensis* obtained from the Protein Data Bank (PDB ID: 4LM8) was aligned with the MtrC component of MtrCAB from *S. baltica*. Finally, the MtrAB complex was combined with the aligned MtrC structure of *S. oneidensis* to assemble the complete MtrCAB complex of *S. oneidensis*.

To build the MtrCAB-membrane complex, we used MtrAB of *S. baltica* in CHARMM-GUI. A pore was generated nearly at the middle of the membrane, into which the MtrAB of *S. baltica* was inserted. Finally, the previously constructed MtrCAB complex of *S. oneidensis* was aligned with the MtrAB complex of *S. baltica*, which was then replaced by MtrCAB of *S.*

oneidensis. The system was solvated with TIP3P water molecules. A total of 155 Na^{+} and 155 Cl^{-} were added to maintain the 0.15 M NaCl concentration in the system. Additionally, the system was neutralized by adding 100 Na^{+}.

```
*********************************************************************************
                                                 36
                                                  ↑
S. oneidensis:   MKNCLKMKNLLPALTITMAMSAVMALVVTPNAYASKWDEKMTPEQVEATLDKKFAEGNYS  60
S. baltica:      -----------------------------------KWDEKMTPEQVEATLDKKFAEGNYS  25

S. oneidensis:   PKGADSCLMCHKKSEKVMDLFKGVHGAIDSSKSPMAGLQCEACHGPLGQHNKGGNEPMIT  120
S. baltica:      PKGADSCLMCHKK----MDLFKGVHGAIDSSKSPMAGLQCEACHGPLGQHNK---EPMIT  78

S. oneidensis:   FGKQSTLSADKQNSVCMSCHQDDKRMSWNGGHHDNADVACASCHQVHVAKDPVLSKNTEM  180
S. baltica:      FGKQSTLSAEKQNSVCMSCHQDDKRVSWNGSHHDNADVACASCHQVHVAKDPVLSKNTEM  138

S. oneidensis:   EVCTSCHTKQKADMNKRSSHPLKWAQMTCSDCHNPHGSMTDSDLNKPSVNDTCYSCHAEK  240
S. baltica:      EVCTSCHTKQKADMNKRSSHPLKWAQMTCSDCHNPHGSMTDSDLNKPSINETCYSCHAEK  198

S. oneidensis:   RGPKLWEHAPVTENCVTCHNPHGSVNDGMLKTRAPQLCQQCHASDGHASNAYLGNTGLGS  300
S. baltica:      RGPKLWEHAPVTENCVTCHNPHGSVNDAMLKTRAPQLCQQCHASDGHASNAYLGNTGLGS  258

S. oneidensis:   NVGDNAFTGGRSCLNCHSQVHGSNHPSGKLLQR 333
S. baltica:      NVGDNAFTGGRSCLNCHSQVHGSNHPSGKLLQR 291
**************************************************
```

Figure S1: Sequence alignment of MtrA between *S. oneidensis* and *S. baltica*. The amino acids highlighted in blue are the residues that are missing in *S. baltica*, while the amino acids highlighted in red are mutated in the structure of MtrA from *S. baltica* based on the amino acid sequence of MtrA from *S. oneidensis*. In this study, the amino acid sequence of MtrA of *S. oneidensis* ranges from residues 36 (shown with an arrow) to 333.

```
************************************************************************************
                                                                       47
                                                                       ↑
S. oneidensis:   MKFKLNLITLALLANTGLAVAADGYGLANANTEKVKLSAWSCKGCVVETGTSGTVGVGVG  60
S. baltica:      ----------------------------------------------VETGTSGTVGVGVG  14

S. oneidensis:   YNSEEDIRSANAFGTSNEVAGKFDADLNFKGEKGYRASVDAYQLGMDGGRLDVNAGKQGQ  120
S. baltica:      YNSEEDIRSANAFGTSNEVAGKLDADVTFRGEKGYRASVEAYQLGMDGGRLEVNAGKQGQ  74

S. oneidensis:   YNVNVNYRQIATYDSNSALSPYAGIGGNNLTLPDNWITAGSSNQMPLLMDSLNALELSLK  180
S. baltica:      YNVNVNYRQIATYNSNSALTPYSGVGSDNLTLPDNWVTAGSSSQMPLLMDSLNSLELSLK  134

S. oneidensis:   RERTGLGFEYQGESLWSTYVNYMREEKTGLKQASGSFFNQSMMLAEPVDYTTDTIEAGVK  240
S. baltica:      RERTGLGFDYQGESLWSTHVSYMREEKTGLKKASGGFFNQSMMLAEPVDYTTDSIEAGIK  194

S. oneidensis:   LKGDRWFTALSYNGSIFKNEYNQLDFENAFNPTFGAQTQGTMALDPDNQSHTVSLMGQYN  300
S. baltica:      LKGDNWFTALNYNGSIFKNEYNQLNFDSAFNPTFGAQTSGSIALDPDNQSHTVSLMGQYN  254

S. oneidensis:   DGSNALSGRILTGQMSQDQALVTDNYRYANQLNTDAVDAKVDLLGMNLKVVSKVSNDLRL  360
S. baltica:      DSTNVLSARLLTGQMSQDQALVTSGYGY--QVPTEALDAKVDLIGLNLKVVSKVTNSLRL  312

S. oneidensis:   TGSYDYYDRDNNTQVEEWTQISINNVNGKVAYNTPYDNRTQRFKVAADYRITRDIKLDGG  420
S. baltica:      SGSYDYNDRDNNTQIEEWTQVSINNVNGKVAYNTPYDNTSQRFKVAADYRITRGMKLDGG  372

S. oneidensis:   YDFKRDQRDYQDRETTDENTVWARLRVNSFDTWDMWVKGSYGNRDGSQYQASEWTSSETN  480
S. baltica:      YDFRRDERNYQDRETTDENTVWARFRVNSFETWDMWVKGSYGQRDGSEYQASEWTSSETN  432

S. oneidensis:   SLLRKYNLADRDRTQVEARITHSPLESLTIDVGARYALDDYTDTVIGLTESKDTSYDANI  540
S. baltica:      SLLRKYNLANRDRTQVEARVTHSPIESLTIDFGARYALDDYTDTVIGLTESKDTSYDANI  492

S. oneidensis:   SYMITADLLATAFYNYQTIESEQAGSSNYSTPTWTGFIEDQVDVVGAGISYNNLLENKLR  600
S. baltica:      SYMITDDLLANAFYNYQIIESEQAGSSNYSTPTWTGFIEDKVDVVGAGISYNNLLENKLR  552

S. oneidensis:   LGLDYTYSNSDSNTQVRQGITGDYGDYFAKVHNINLYAQYQATEKLALRFDYKIENYKDN  660
S. baltica:      MGLDYTYSDSNSNTQVRQGITGDYGDYFAKVHNINLYAQYQATEKMALRFDYKIENYKDN  612

S. oneidensis:   DAANDIAVDGIWNVVGFGSNSHDYTAQMLMLSMSYKL   697
S. baltica:      DAANDIAVNGIWNVVGFGDNSHDYTAQMIMLSMSYKI   649
*************************************************************
```

Figure S2: Sequence alignment of MtrB between *S. oneidensis* and *S. baltica*. The color code are same as in Figure S1. In addition, the amino acids highlighted in purple are extra residues found in *S. oneidensis*, but not present in *S. baltica*. In this study, the amino acid sequence in MtrB of *S. oneidensis* ranges from residues 47 (shown with an arrow) to 697.

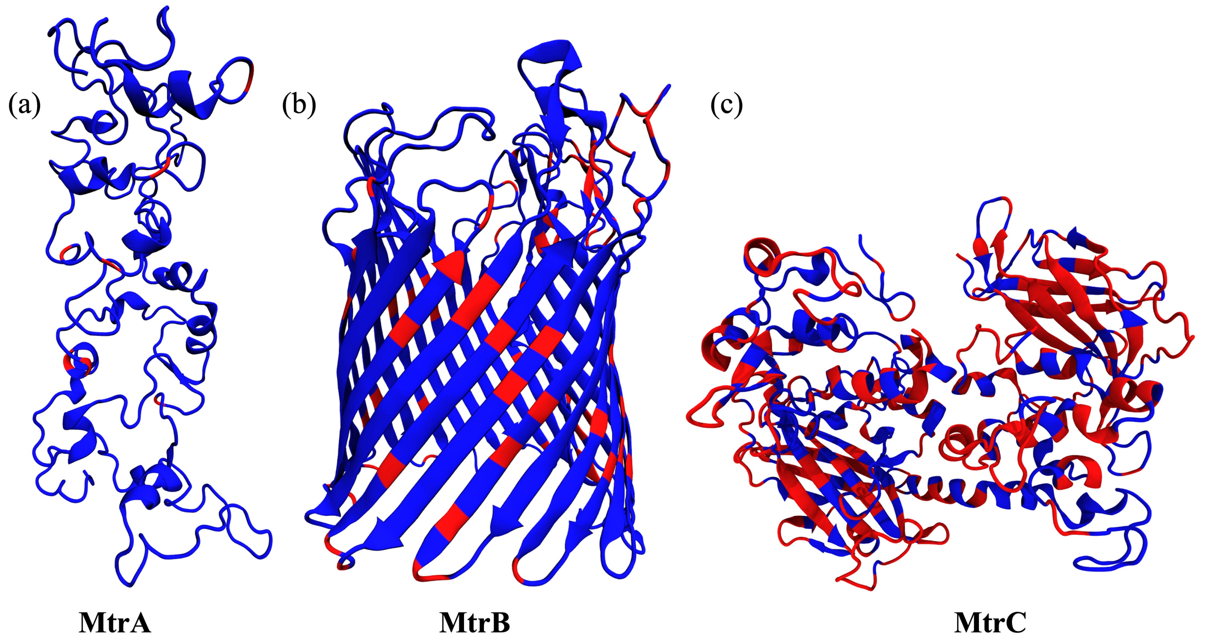

Figure S3: Sequence similarity between *S. oneidensis* and *S. baltica* projected on (a) MtrA, (b) MtrB, and (c) MtrC. The residues in red illustrate mismatch residues, while the color blue denotes the residues common to both *S. oneidensis* and *S. baltica*.

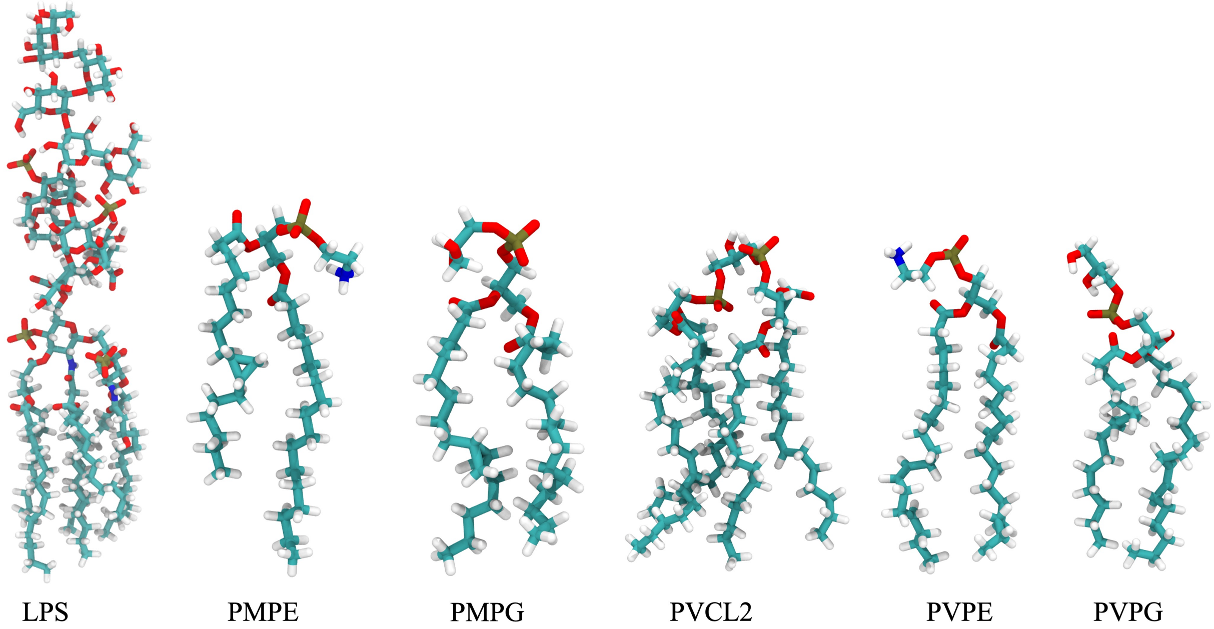

Figure S4: Structures of different lipids used in the OM model. Phosphorous, oxygen, nitrogen, carbon, and hydrogen atoms are shown in tan, red, blue, cyan, and white, respectively.

Comparison of equilibrium MD simulations and REMD simulations results

We calculated root mean square fluctuations (RMSF) of the MtrA, MtrB, and MtrC from equilibrium simulations in the presence and the absence of the calcium ions for both the oxidation states (Figure S5a-f). A detailed discussion on RMSF from equilibrium simulations is provided in a later section “Root mean square fluctuations (RMSF) of the residues of MtrCAB”. We show the RMSF of MtrA, MtrB, and MtrC from REMD simulations in Figure S5g-i. The RMSF values of the residues belonging to the highly flexible regions in the interface of MtrA-MtrC and MtrB-MtrC, calculated from both equilibrium MD and replica exchange molecular dynamics (REMD) simulations, are shown in Figure S6. Except for very few residues, the RMSF values of these residues do not significantly change between the equilibrium and the REMD simulations.

We quantified interfacial residues within 5 Å of the MtrA-MtrC and MtrB-MtrC interfaces for the following systems: MtrCAB from *S. baltica* crystal structure, 1 ns equilibrated structure from *S. oneidensis*, average from 3.3 μs equilibrium and average from 162 ns REMD simulations from *S. oneidensis*. The results are presented in Table S2. The MtrA-MtrC interface in the equilibrium structure of *S. oneidensis* contains five more residues than in *S. baltica*, whereas the number of residues in the MtrB-MtrC interface remains comparable between the two species. The average number of residues in the MtrA-MtrC interface, as determined from equilibrium MD simulations, is similar to that in *S. baltica*. However, the equilibrium simulations reveal an increase of ten residues in the MtrB-MtrC interface. Furthermore, REMD simulation reveals two additional residues at the MtrA-MtrC interface and four fewer residues at the MtrB-MtrC interface compared to the 3.3 μs equilibrium simulations.

Overall, our results suggest that the interfaces between MtrA and MtrC, as well as MtrB and MtrC in *S. oneidensis* remain stable. We show the interface of MtrCAB from the crystal structure of *S. baltica* and the 1 ns equilibrated structure of *S. oneidensis*, superimposed onto

each other in Figure S7.

Root mean square fluctuations (RMSF) of the residues of MtrCAB

We calculated RMSF of each residue of MtrA, MtrB, and MtrC in the presence and absence of calcium ions for both the oxidation states from our MD simulation trajectories, shown in Figure S5. The polypeptide chain of MtrA is predominantly composed of flexible loops (80%) with a smaller proportion of helices (20%).[S3,S4] Since the tail region (N-terminal) of MtrA is located in the periplasm, the residues in this region exhibit higher fluctuations both in the presence and absence of calcium ions of both oxidation states. These are shown in Figures S5a,b for the oxidized and reduced states, respectively. The remainder of MtrA, located within the β-barrel, shows constrained fluctuations due to the presence of MtrB, as observed in our RMSF. The β-sheets within the β-barrel protein embedded in the outer membrane (OM) of Gram-negative bacteria are stabilized by strong hydrogen bonding, resulting in a stable β-barrel structure.[S5] Consequently, we observe smaller RMSF values of most residues in MtrB (Figures S5c,d) for all cases.

Figures S5e,f shows the root mean square fluctuation of the MtrC in the MtrCAB complex. A particular domain of the MtrC, consisting of residues 45-175 and referred to as "D1" exhibits higher fluctuations for all cases. A similar finding is also observed in a previous study on MtrC, which reported that D1 is less stable than the rest of the MtrC.[S6] We have highlighted this D1 domain in Figure S14.

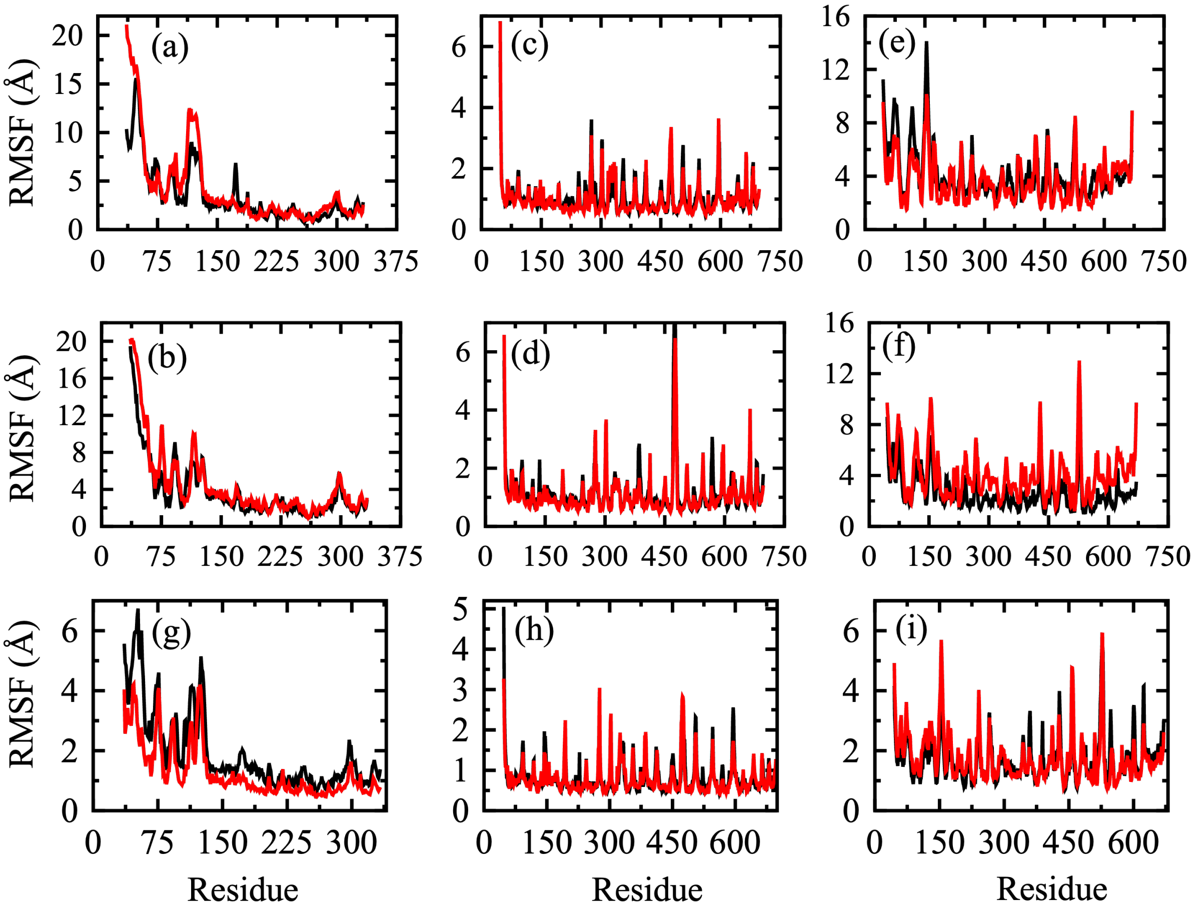

Figure S5: Root mean square fluctuation (RMSF) of the residues in MtrA for (a) oxidized and (b) reduced states. RMSF of the residues of MtrB in (c) oxidized and (d) reduced states. Panels (e) and (f) illustrate the same as (a) and (b), respectively, but for MtrC. (g), (h) and (i) illustrate the RMSF for MtrA, MtrB, and MtrC, respectively from REMD simulations. The results for the presence and absence of calcium ions are illustrated in black and red, respectively. The RMSF has been averaged over 3.3 μs simulations for each residue for both oxidation states. The RMSF shown in (g), (h), and (i) are averaged over 162 ns REMD simulations.

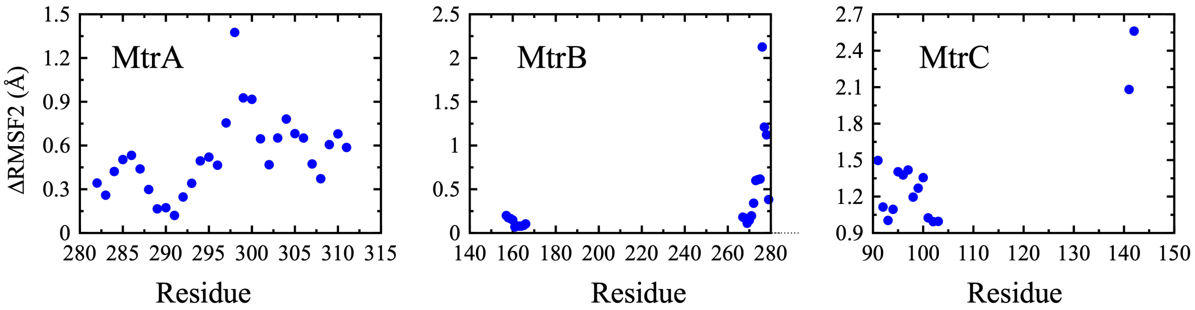

Figure S6: Change in RMSF (ΔRMSF2 (=RMSF (MD) - RMSF (REMD))) between equilibrium MD and REMD simulations in the oxidized states for the residues in MtrA, MtrB, and MtrC at the protein-protein interface in the presence of the calcium ions.

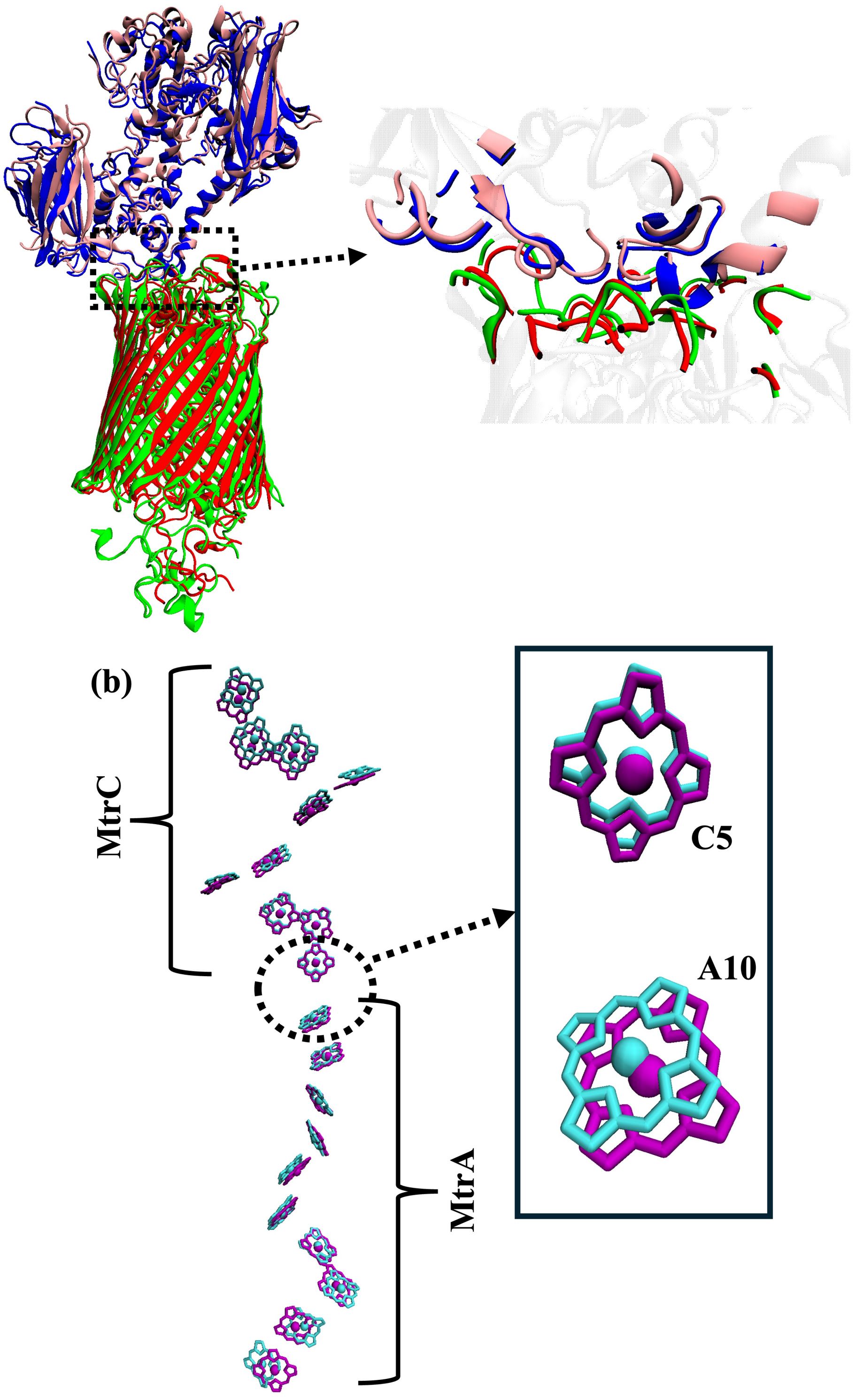

Figure S7: (a) Superposition of the full MtrCAB complex from the 1 ns equilibrated structure of MtrCAB from *S. oneidensis* and from the crystal structure of MtrCAB from *S. baltica* (left) and a zoomed-in view of the protein-protein interface (right). MtrAB and MtrC for *S. oneidensis* are illustrated in green and pink, respectively, while those for *S. baltica* are represented as red and blue, respectively. (b) The heme chain (left) and the zoomed-in view of the interfacial hemes (right) for *S. oneidensis* (cyan) and *S. baltica* (purple). The alignment of MtrCAB between the two species was performed based on the C-α atoms of MtrB.

Stability of MtrB-bound Ca^{2+} ions

Two calcium ions are located on the surface of MtrB β-barrel. We determined the coordination geometry of calcium ions in the oxidized and reduced states and compared it with the available crystal structure data. The coordination of MtrB-bound calcium ions from the crystal structure is shown in Figure S8a,b, illustrating the first and second calcium ions, respectively. In the crystal structure, the first calcium ion is coordinated with the main chain of ASN259 and ASN262, and the side chain of ASP287, while the second calcium ion is coordinated with the side chains of ASP370 and ASP397, and the main chain of ASN371 (Table S3). We also calculate the coordination of calcium ions and their occupancies from our MD simulation trajectories in both oxidation states (Table S3). The snapshots showing the coordination of the first calcium ion from the simulated structure of the oxidized and the reduced states are in Figures S8c,d, respectively. Our MD simulation trajectory shows that the residue ASN259 is loosely coordinated with the first calcium ion in the oxidized state (occupancy 33%). In contrast, this occupancy increases to 73% in the reduced state. We also observe that ASN262 leaves the binding site, and the side chain of GLU260 coordinates with the first calcium ion in both oxidation states with a similar occupancy. The residue ASP287 remains coordinated with the first calcium ion throughout the simulations in both oxidation states. The coordination of the second calcium ion in oxidized and reduced states remains the same as in the crystal structure. Overall, the calcium ions remain stably coordinated at the MtrB binding site throughout the simulations, consistent with their positioning in the crystal structure.

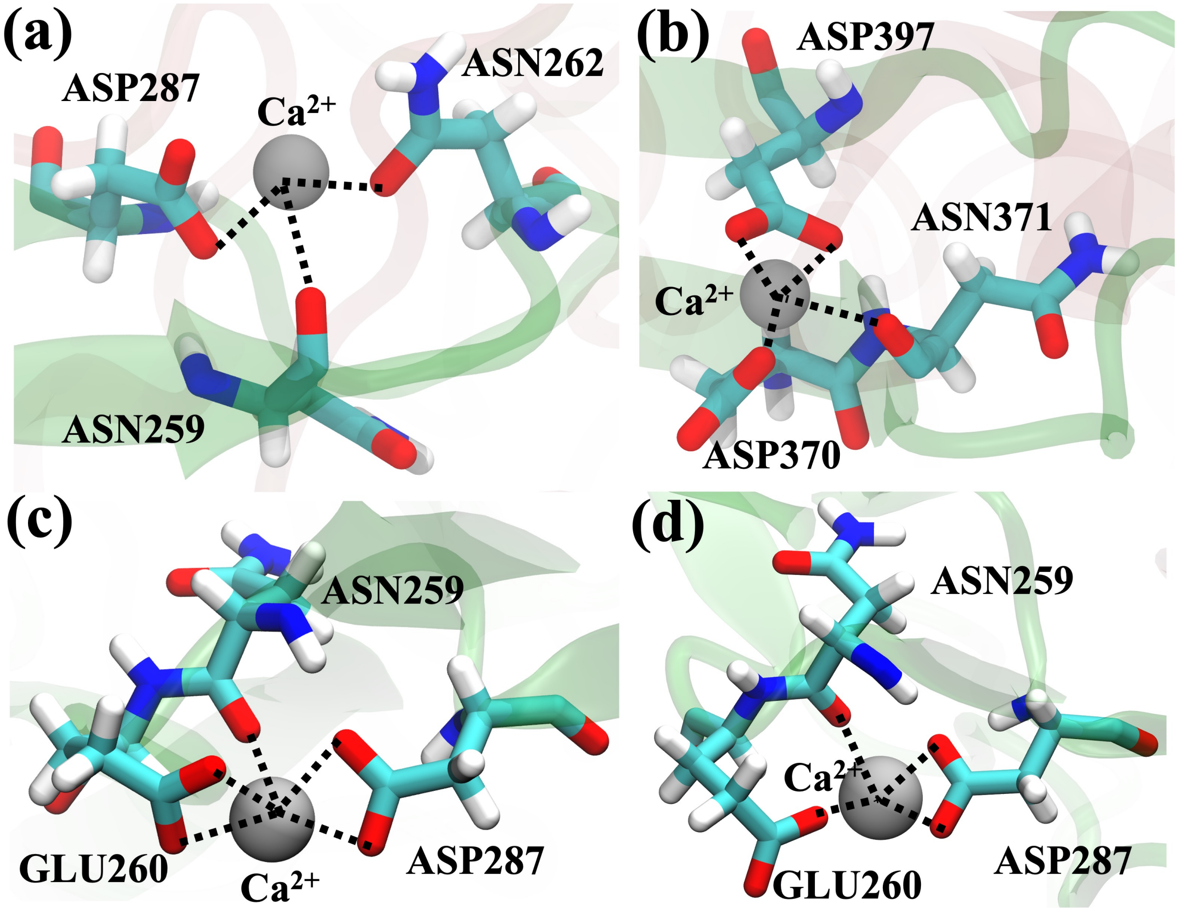

Figure S8: Coordination of the (a) first and (b) second calcium ions in the initial structure of MtrCAB of *S. oneidensis*. Snapshots showing the coordination of the first calcium ion from the simulated structures for (c) the oxidized and (d) the reduced states. The coordination of the second calcium ion from the simulated trajectory is not shown, as it remains consistent with the coordination observed in the crystal structure.

Sequence alignment of MtrB between different *Shewanella* species

To investigate the importance of calcium-coordinating residues across different *Shewanella* species, we analyzed the sequences of MtrB from seven other representative *Shewanella* species, *Shewanella decolorationis*, *Shewanella frigidimarina*, *Shewanella gelidii*, *Shewanella sp.* HN-41, *Shewanella sp.* MR-7, *Shewanella sp.* ANA-3, and *Shewanella sp.* LC6. The sequence alignment of the residues in MtrB for all these *Shewanella* species was performed based on the MtrB sequence of *Shewanella oneidensis* (Figure S9). Our analysis revealed that, with the exception of *Shewanella frigidimarina* and *Shewanella gelidii*, these calcium-coordinating residues are highly conserved across the other five species. In *S. frigidimarina*, ALA262 is mutated by GLY, and ASP371 is mutated by GLU. However, this variation may not significantly impact calcium coordination as GLU maintains coordination through its side chain, similar to ASP371 in other species, while GLY still participates in coordination via backbone interactions. These findings indicate that the calcium-binding mechanism is likely preserved in *S. frigidimarina* despite minor sequence variations. In case of *Shewanella gelidii*, both GLU260 and ALA371 are mutated by ASP. Mutation of ALA371 to ASP in this species, makes the calcium coordination stronger. Overall, the high conservation of calcium-coordinating residues across these *Shewanella* species suggests that calcium ions may play a functional role in facilitating electron transfer in these organisms.

```
                                                                 259 260 262                          287
S. oneidensis MR-1      VDYTTDTIEAGVKLKGDRWFTALSYNGSIFKNEYNQLDFENAFNPTFGAQTQGTMALDPDNQSHTVSLMGQYNDGSNALS  307
S. decolorationis       VDYTTDTIEAGVKLKGDRWFTALSYNGSIFKNEYNQLNFESAFNPTFGAQTQGTMALDPDNQAHTVSLMGQYNDGSNSLS  315
S. frigidimarina        VDYTTDILNAGIKLRGDNWFTSINYNGSVFSNNNQQLGFDSAFNPTFGAQSRGYMALDPDNEAHTISLMGQYNDSITSLS  307
S. gelidii              VDTSTDQLNAGLNLHGQNWFANLSYTGSKFDNDHNQLGYDNLFNPTFGAQTTGYMALDPDNESHQFALQARYMLDNTVFS  312
Shewanella sp. HN-41    VDYTTDTIEAGVKLKGDRWFTALNYNGSIFKNEYNQLNFDSAFNPTFGAQTSGAIALDPDNQSHTVTLMGQYNDSTNVLS  307
Shewanella sp. LC6      VDYTTDTIEAGVKLKGDRWFTALSYSGSIFKNEYNQLDFDSAFNPTFGAQTQGTLALDPDNQAHTVSLMGQYNDGSNALS  307
Shewanella sp. ANA-3    VDYTTDTIEAGVKLKGDRWFTALSYNGSIFKNEYNQLNFDSAFNPTFGAQTSGAIALDPDNQSHTVSLMGQYNDGSNSLS  307
Shewanella sp. MR-7     VDYTTDTIEAGVKLKGDRWFTALSYNGSIFKNEYNQLNFDSAFNPTFGAQTTGAIALDPDNQSHTVSLMGQYNDGSNSLS  307

                                                                                            370 371
S. oneidensis MR-1      GRILTGQMSQDQALVTDNYRYANQLNTDAVDAKVDLLGMNLKVVSKVSNDLRLTGSYDYYDRDNNTQVEEWTQISINNVN  387
S. decolorationis       GRILTGQMSQDQALVTDNYRYANQLNTDAVDAKVDLLGMNLKVVSKVSKDLRLTGSYDYYDRDNNTQVEEWTQISINNVN  395
S. frigidimarina        GRLLLGQMTQDQQLTSIGYGYS--LPADSIDAKVDITGMTLKAVTKLNRAWRLTGSYDYNDRENNTQIEQWTQISINDVT  385
S. gelidii              GRLHYANMTQDQDFVQMGYLYP--MPVPSLDGQVDVSGLTLKATTRMSRALQVNVSYDYYSRDDDTQVEAWTQYTVSDVN  390
Shewanella sp. HN-41    GRILAGQMSQDQALVTSGYGY--QVPTEAVDAKVDLLGMNLKVVSKVSNSVRLTGSYDYHDRDNNTQIEEWTQISINNVN  385
Shewanella sp. LC6      GRILTGQMSQDQALVTDNYRYSSQLTSDAVDAKVDLLGMNLKVVSKVSKDLRLTGSYDYYDRDNNTQVEEWTQISINNVN  387
Shewanella sp. ANA-3    GRILTGQMSQDQSLVTSGYGY--QLPTDAVDAKVDLLGMNLKVVSKVSNSLRLSGSYDYYDRDNNTPIEEWTQISINNVS  385
Shewanella sp. MR-7     GRILTGQMSQDQSLVTSGYGY--QLPTDAVDAKVDLLGMNLKVVSKVSNSLRLTGSYDYYDRDNNTPIEEWTQISINNVS  385

                                  397
S. oneidensis MR-1      GKVAYNTPYDNRTQRFKVAADYRITRDIKLDGGYDFKRDQRDYQDRETTDENTVWARLRVNSFDTWDMWVKGSYGNRDGS  467
S. decolorationis       GKVAYNTPYDNRTQRFKVAADYRITRDIKLDGGYDFKRDQRDYQDRETTDENTVWARLRVNSFDTWDMWVKGSYGNRDGS  475
S. frigidimarina        GKVRYNTPYDLTTHNAKLSTDYRINHGLKLDAGYDFKRDERSNQGRETTDDNNVWARLRVNSFDMWDMWLKGSLSKRDGS  465
S. gelidii              GEIYYNLPYDHDKHLAKISANYRLARGMKLEAGLDYRKDERNYQDRETTDEQTLWAKYSLTNIPMWNFYLKADYGQRDGS  470
Shewanella sp. HN-41    GKVAYNTPYDNRSQRFKVAADYRITHGMKLDGGYDFKRDQRDYQDRETTDENTVWARFRVNSFDMWDMWVKGSYANRDGS  465
Shewanella sp. LC6      GKVAYNTPYDNRTQRFKVAADYRITRDIKLDGGYDFKRDQRDYQDRETTDENTVWARLRVNSFDMWDMWVKGSYGKRDGS  467
Shewanella sp. ANA-3    GKVAYNTPYDNRTQRFKVAADYRITHGIKLDGGYDFKRDEREYQDRESTDENTVWARLRVNSFDMWDMWVKGSYGNRDGS  465
Shewanella sp. MR-7     GKVAYNTPYDNRTQRFKVAADYRITHGIKLDGGYDFKRDEREYQDRESTDENTVWARLRVNSFDMWDMWVKGSYGNRDGS  465
```

Figure S9: Sequence alignment of MtrB between different species of *Shewanella*. Calcium-coordinating residues are illustrated in green and red. The residues in green represent those that are conserved across all species, whereas the residues in red indicate differences between any two *Shewanella* species. The numbering of calcium-coordinating residues is based on the MtrB of *S. oneidensis*. Except for GLU260, all other marked residues are coordinated with calcium ions in the crystal structure of MtrCAB of *S. oneidensis*, while in addition to those residues, GLU260 is also coordinated with one of the calcium ions in the simulated structure. The uniprot IDs of the sequence of MtrB for *S. oneidensis* MR-1, *S. decolorationis*, *S. frigidimarina*, *S. gelidii*, *Shewanella* sp. HN-41, *Shewanella* sp. LC6, *Shewanella* sp. ANA-3, and *Shewanella* sp. MR-7 are Q8CVD4, A0A5B8QZS8, Q84EK5, A0A917JLF8, F7RME5, A0A501Y2U3, A0KYN6, and Q0HTJ0, respectively. The sequence alignment was performed using COBALT.[S7] Since we are only interested in calcium-coordinating residues, the full MtrB sequence has not been shown here for any species.

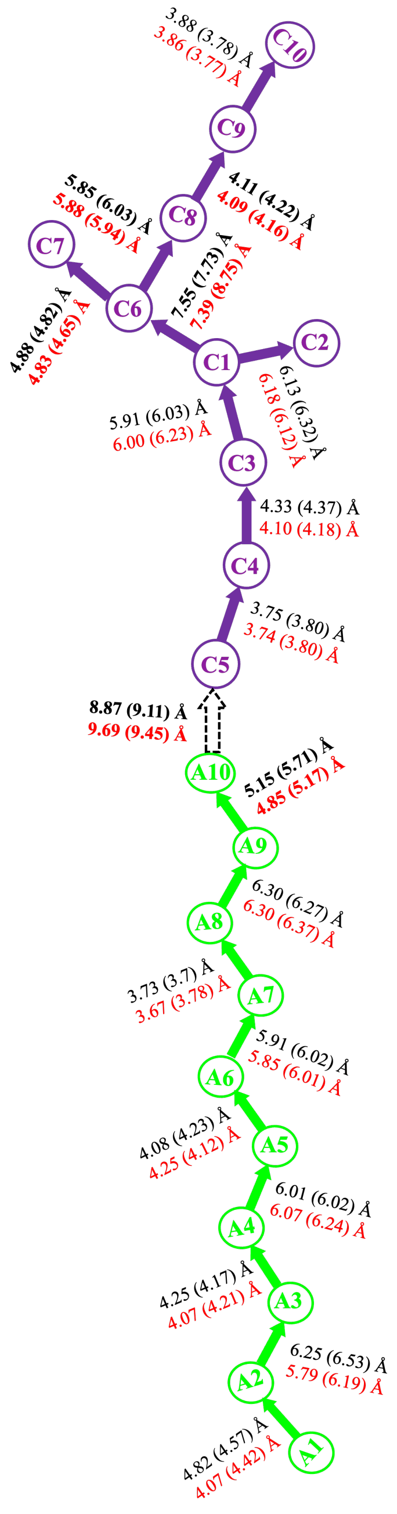

Figure S10: Heme-heme distances of each heme pair in the MtrCAB complex in the presence (black) and absence (red) of calcium ions. The values outside the parenthesis illustrate the heme-heme distance in the oxidized state and the values within represent the distances in the reduced state. The data were averaged over 3.3 μs simulations.

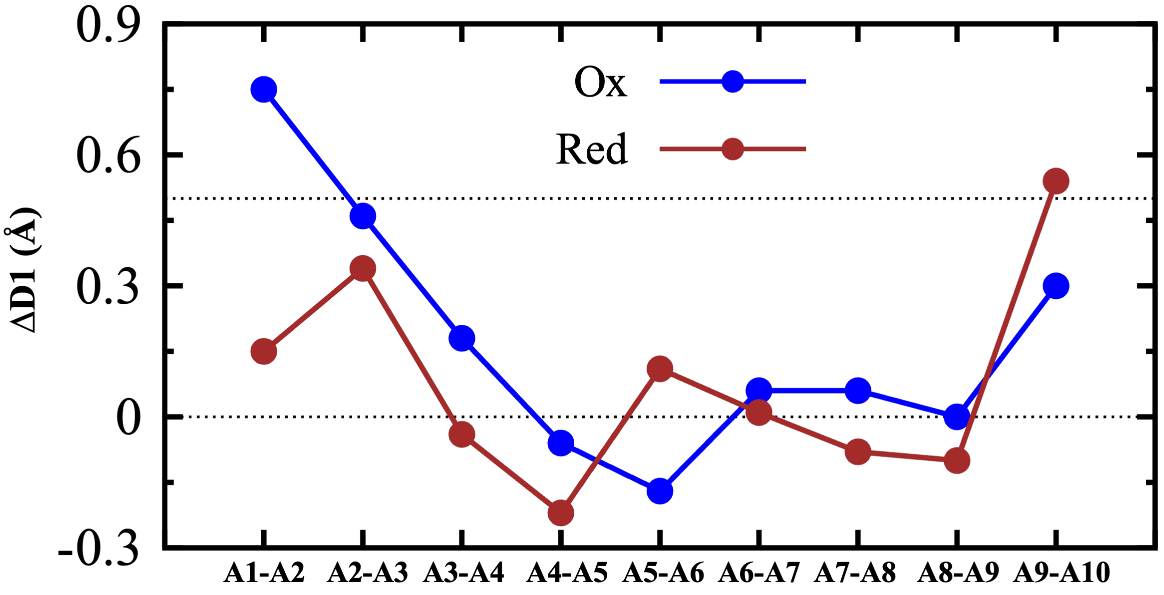

Figure S11: Heme-heme distance difference (ΔD1=Distance ($+Ca^{2+}$) - Distance ($-Ca^{2+}$)) between in presence and absence of calcium ions for the oxidized state (blue) and the reduced state (brown), averaged over 3.3 μs simulations.

Electron transfer rate estimation

We estimated electron transfer rates using Moser-Dutton ruler.[S8]

$\log k_{et} = 15 - 0.6R - 3.1(\Delta G - \lambda)^2/\lambda$

Where k_{et} is the electron transfer rate constant, R is the heme-heme distance, ΔG is the Gibbs free energy, and λ is the reorganization energy. We calculated k_{et} in the presence (k_{et}^{+Ca}) and absence (k_{et}^{-Ca}) of calcium ions for the heme pairs in which the heme-heme distances are equal to or greater than 0.5 Å. We considered the values of ΔG and λ for a particular heme pair the same in both the presence and absence of calcium ions. Finally, we determined the ratio of electron transfer rate constants in the presence and absence of calcium ions ($k_{et}^{+Ca}/k_{et}^{-Ca}$).

We calculated the heme-heme distances within MtrA in the presence and absence of calcium ions for both oxidation states (Figure S11). All the values of ΔD1=(Distance ($+Ca^{2+}$) - Distance ($-Ca^{2+}$)) for the heme groups within MtrA are minimal with a deviation of $\sim$

± 0.35 Å. However, the distance increases by 0.75 Å ($k_{et}^{+Ca}/k_{et}^{-Ca}$ = 2.86) for A1-A2 and by 0.54 Å ($k_{et}^{+Ca}/k_{et}^{-Ca}$ = 2.13) for A9-A10, both in the presence of calcium ions compared to those in the absence of calcium ions. The heme pair A1-A2 belongs to the periplasmic region and is exposed to water. Therefore, larger fluctuations are expected for this region. A loop of MtrB containing residues ALA471-SER476, located near heme A9, exhibits increased ΔRMSF values, particularly for residues SER472, GLU473, and TRP474 with ΔRMSF ranging from 2.5 to 3 Å (Figure S12 and S13). This leads to an increase in the A9-A10 distance.

RMSF difference (ΔRMSF = RMSF ($+Ca^{2+}$) - RMSF ($-Ca^{2+}$))

To gain a deeper understanding of the impact of calcium ions on the fluctuations of the residues, we calculated difference in RMSF (ΔRMSF = RMSF ($+Ca^{2+}$) - RMSF ($-Ca^{2+}$)) for each residue between simulations in the presence and absence of calcium ions (Figure S13). The positive value of ΔRMSF indicates that the RMSF is higher in the presence of calcium ions, while the negative value means the RMSF is higher in the absence of calcium ions. We separately projected ΔRMSF values onto MtrA, MtrB, and MtrC, as shown in Figures S12a-c for the oxidized state and Figures S12d-f for the reduced state. In both oxidation states, most residues belonging to the tail region (N-terminus) show larger fluctuations in the absence of the calcium ions. However, a few residues in this region exhibit higher fluctuations in the presence of calcium ions. Fluctuations of the other residues (residues 176 to 333) of MtrA are similar in both oxidation states. The data are shown in Figure S13a,b for the oxidized and reduced states, respectively. The ΔRMSF values for the residues of MtrB in the reduced state are negligible between the simulations of the presence and absence of the calcium ions (Figure S13c). However, only very few residues show increased fluctuation in the presence of the calcium ions in the reduced state (Figure S13d). Most of the residues of MtrC belonging to domain D1 (Figure S13e) show higher fluctuations in the presence of calcium ions in the oxidized state. However, this D1 domain does not contain any hemes. So, the distances

between the heme groups are expected not to be affected by the fluctuation of the residues belonging to D1. The other residues of MtrC do not exhibit any significant changes between the presence and absence of calcium ions. For the reduced state, unlike in the oxidized state, most of the residues of MtrC show higher fluctuations in the absence of the calcium ions, as shown in Figure S13f.

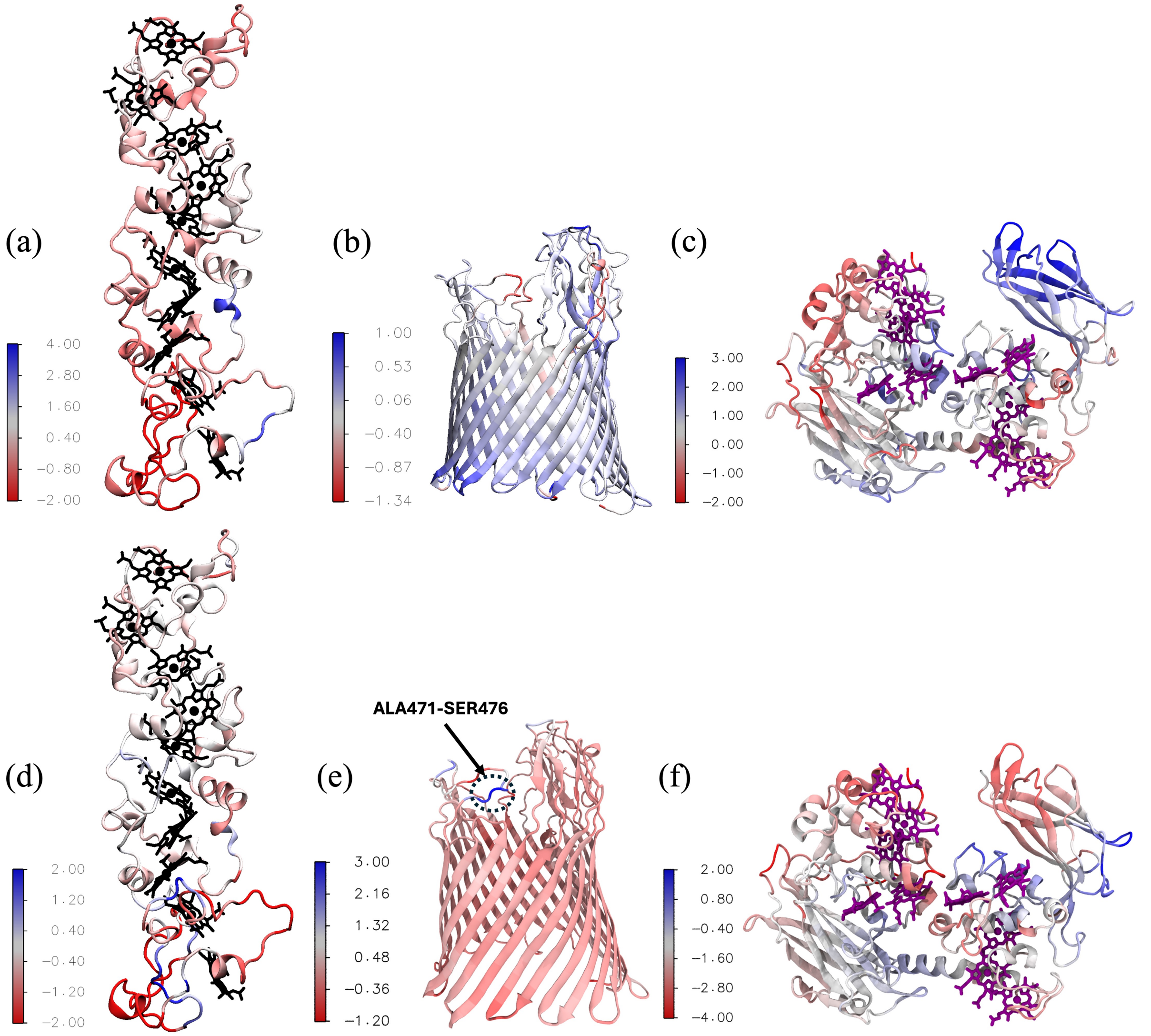

Figure S12: ΔRMSF (= RMSF ($+Ca$) - RMSF ($-Ca$)) projected on (a) MtrA, (b) MtrB, and (c) MtrC of oxidized state and (d) MtrA, (e) MtrB, and (f) MtrC of the reduced state. The RMSF data were averaged over 3.3 μs simulations.

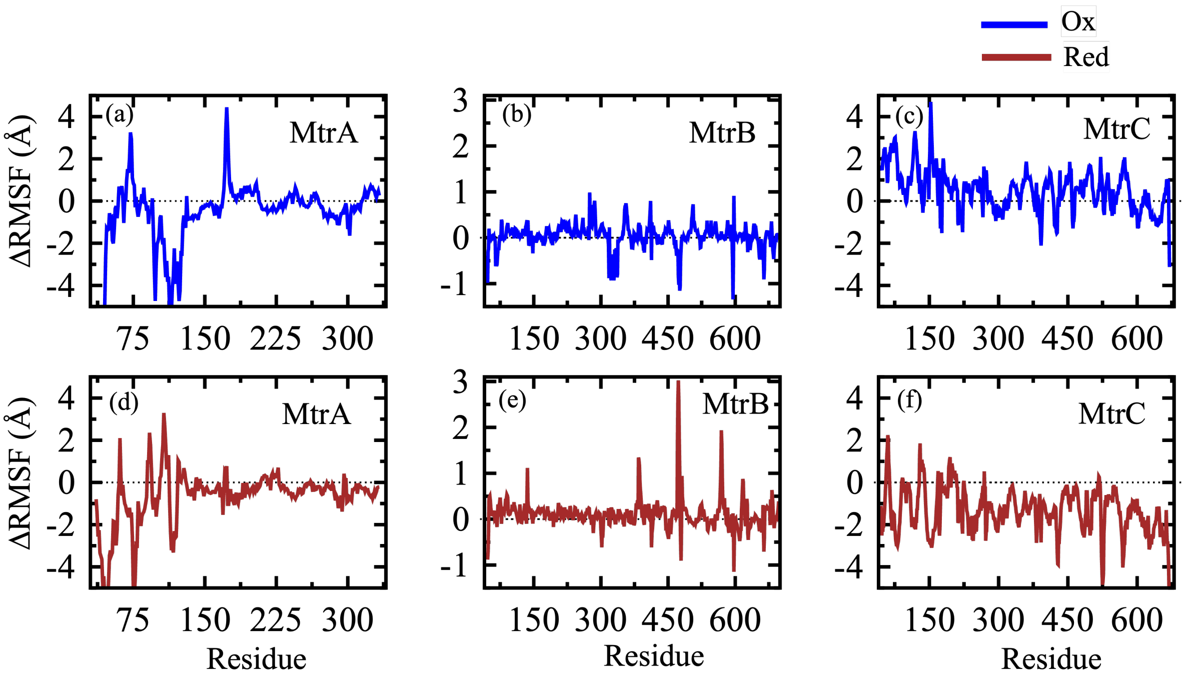

Figure S13: ΔRMSF (= RMSF ($+Ca$) - RMSF ($-Ca$)) of the residues in (a) MtrA, (b) MtrB, and, (c) MtrC between in the presence and absence of calcium ions in the oxidized state. Panels (c), (d), and (e) represent the same as (a), (b), and (c), respectively, but for the reduced state. The RMSF data were averaged over 3.3 μs equilibrium simulations.

Free MtrC simulations and analysis

Among the 3 replicates of the oxidized free MtrC production run, we observed that a particular domain of the protein, consisting of residue 45–175 and referred to as "Domain D1" in Figure S14, unraveled from the rest of the system and then joined back in a different conformation. This observation was unique to a single run of the free MtrC, and therefore we calculated the distance between the center of mass of Domain D1 and the rest of the system. Figure S15 shows that for reduced MtrC and MtrCAB, the average distance between the center of masses of domain D1 and the rest of the system is around 42 Å , similar to the oxidized MtrCAB system. However, for oxidized free MtrC the first production replica shows that around 0.2 μs the D1 domain starts detaching from the MtrC complex and attaches back after 0.4 μs, in a different conformation. Comparing domain D1 between free MtrC and OM-MtrCAB complex, we postulate that the presence of the MtrB and the OM must prevent the detachment observed in the free MtrC simulation. Since this behavior was observed only in one replica of the oxidized free MtrC system, we excluded the frames where domain D1 was detached from the MtrC while calculating heme-heme distances.

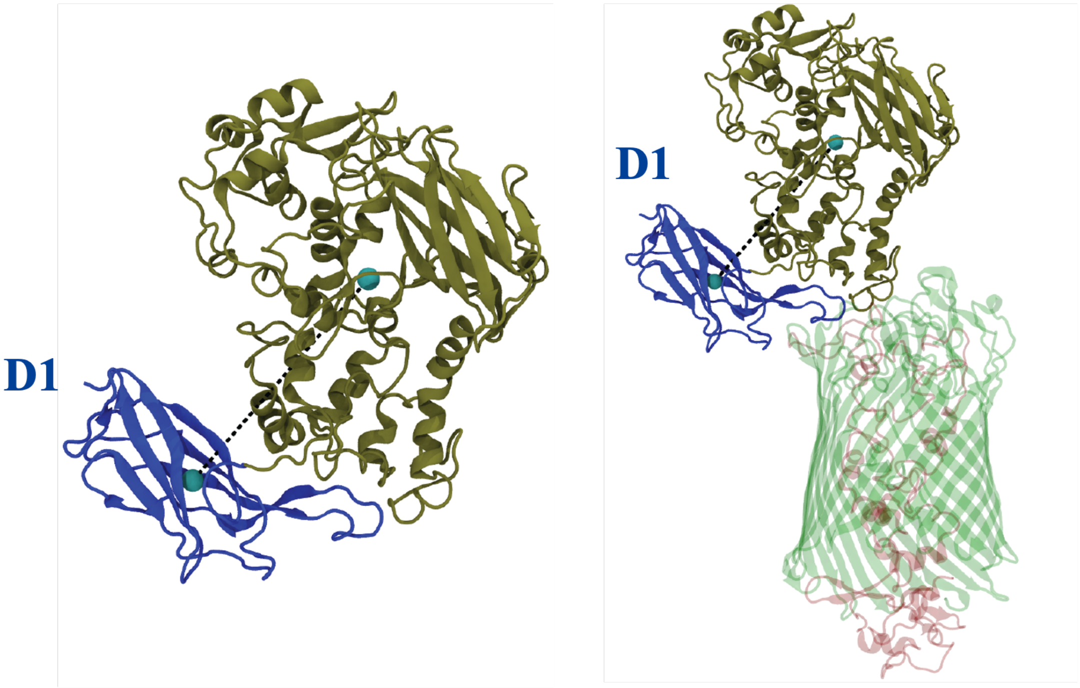

Figure S14: Free MtrC (left) and MtrCAB complex (right) with the domain, D1 (in blue), and the rest of MtrC (in tan). The cyan spheres represent the center of masses of the residues belonging to D1 and the rest of the MtrC system.

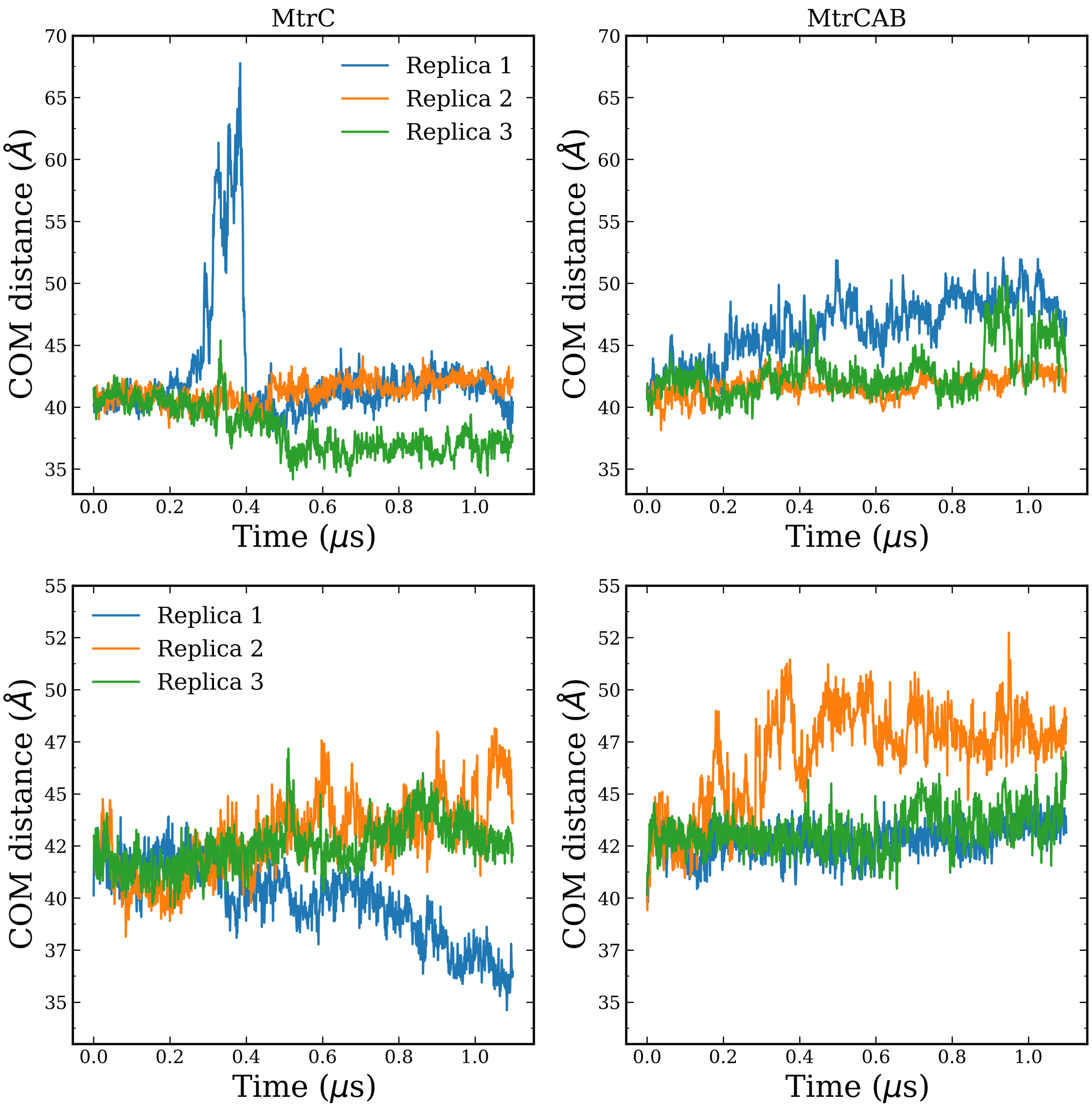

Figure S15: Distance between center of masses (COM) of D1 and the rest of the MtrC for each replica run (1.2 μs). The top and the bottom rows show the data for the reduced and oxidized MtrC for free and from MtrCAB, respectively.

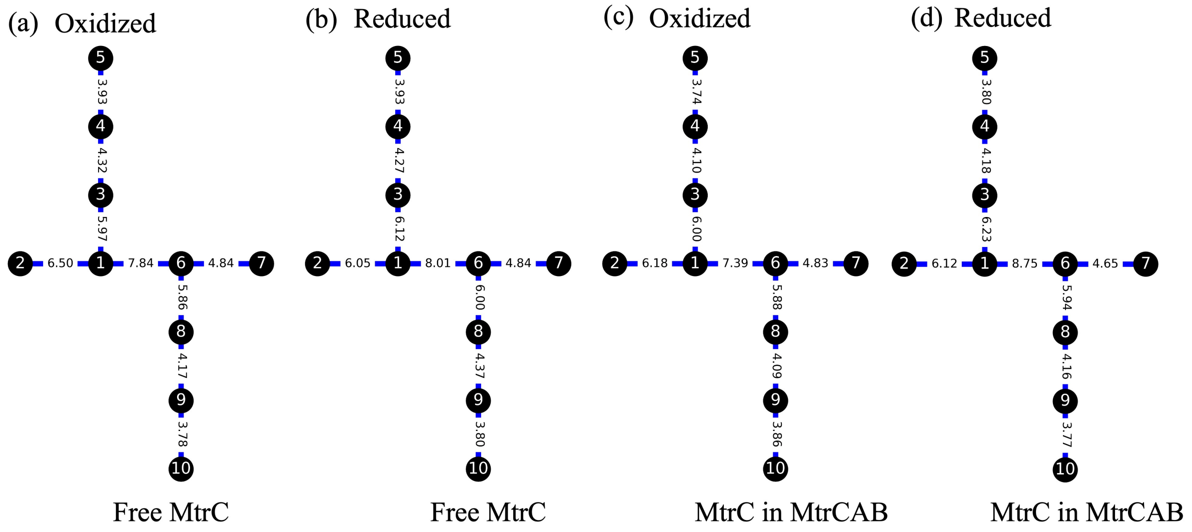

Figure S16: Heme-heme distances for free MtrC in (a) oxidized and (b) reduced states. Heme-heme distances for MtrC in MtrCAB in (c) oxidized and (d) reduced states. Here, the minimum distance between the porphyrin rings of two heme groups was calculated. The distances were averaged over 3.3 μs simulations of each case.

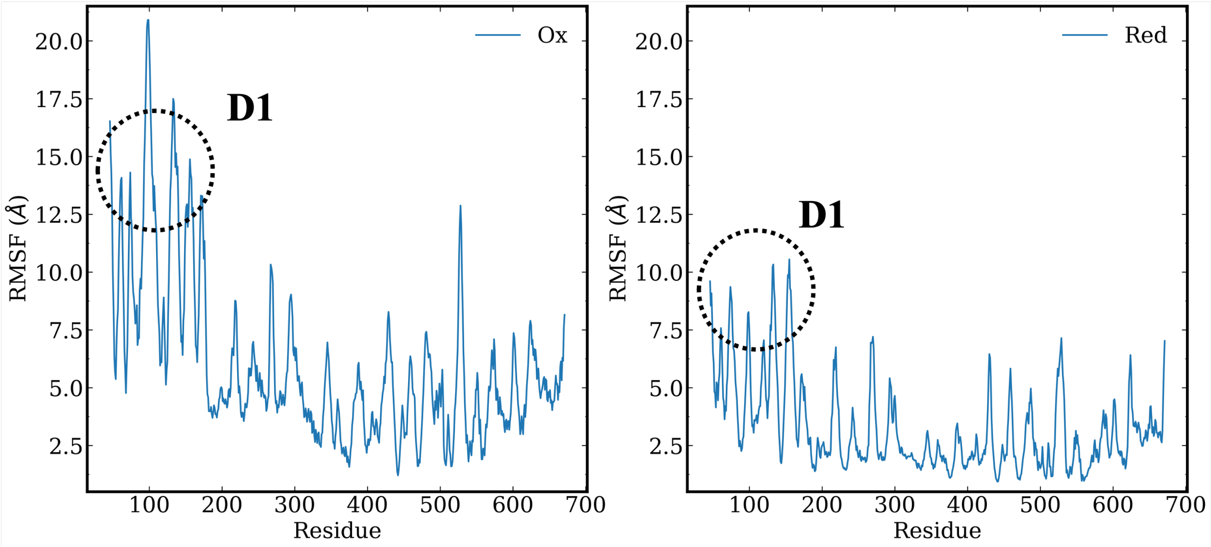

Figure S17: Root mean square fluctuation (RMSF) of the oxidized (left) and reduced (right) free MtrC.

Table S1: System information. UL - Upper Leaflet, LL - Lower Leaflet, Ox - Oxidized, Red - Reduced.

System	LPS UL(LL)	PMPE UL(LL)	PMPG UL(LL)	PVCL2 UL(LL)	PVPE UL(LL)	PVPG UL(LL)	Total no. of water	Total no. of atoms
OM-MtrCAB(Ox)	79 (0)	0 (80)	0 (10)	0 (20)	0 (80)	0 (20)	339180	436303
OM-MtrCAB-Ca(Ox)	79 (0)	0 (80)	0 (10)	0 (20)	0 (80)	0 (20)	339192	436313
OM-MtrCAB(Red)	79 (0)	0 (80)	0 (10)	0 (20)	0 (80)	0 (20)	339120	436263
OM-MtrCAB-Ca(Red)	79 (0)	0 (80)	0 (10)	0 (20)	0 (80)	0 (20)	339132	436273

Table S2: Total number of residues at the MtrA-MtrC and MtrB-MtrC interfaces. The calculations were done based on the total number of residues within 5 Å at MtrA-MtrC and MtrB-MtrC interfaces. The data were averaged over 3.3 μs for the equilibrium MD simulations and 162 ns for the REMD simulations in the oxidized state and in the presence of the calcium ions.

System	No. of residues at the interface of	
	MtrA-MtrC	MtrB-MtrC
S. baltica (Crystal structure)	37	49
S. oneidensis (Equilibrated structure)	42	48
S. oneidensis (Averaged over 3.3 μs MD simulations $\pm$ S. D.)	36 $\pm$ 3	58 $\pm$ 6
S. oneidensis (Averaged over 162 ns REMD simulations $\pm$ S. D.)	38 $\pm$ 2.32	54 $\pm$ 3.86

Table S3: Coordination of calcium ions from the initial structure of MtrCAB from *S. oneidensis* and 3.3 μs simulations for both the oxidized and reduced states.

System	Coordinating residues with occupancy			
	1st calcium		2nd calcium	
	main chain	side chain	main chain	side chain
Crystal structure	ASN259	ASP287	ASN371	ASP370 and ASP397
Simulated structure (Ox)	ASN259 (33%)	ASP287 (100%), GLU260 (86%)	ASN371 (92%)	ASP370 (100%), ASP397 (100%)
Simulated structure (Red)	ASN259 (73%)	ASN262 (4%), ASP287 (100%), GLU260 (73%)	ASN371 (89%)	ASP370 (100%), ASP397 (100%)

Table S4: Heme-heme distances of each heme pair, along with their respective arrangement types within MtrA, MtrC, and between MtrA and MtrC. The data represent an average over 3.3 μs equilibrium MD simulations.

Heme pairs	Arrang-ement	Distance (Oxidation state) (Å)							
		$+Ca^{2+}$ (Ox)	Avg. (Ox)	$-Ca^{2+}$ (Ox)	Avg. (Ox)	$+Ca^{2+}$ (Red)	Avg. (Red)	$-Ca^{2+}$ (Red)	Avg. (Red)
A1-A2	Stacked	4.94		4.06		4.76		4.37	
A3-A4	Stacked	4.19		4.09		4.04		4.18	
A5-A6	Stacked	4.11	4.42	4.33	4.2	4.29	4.43	4.20	4.32
A7-A8	Stacked	3.73		3.65		3.70		3.78	
A9-A10	Stacked	5.15		4.89		5.38		5.06	
A2-A3	T-shaped	6.21		5.85		6.28		6.24	
A4-A5	T-shaped	5.96		6.04		6.05		6.26	
A6-A7	T-shaped	5.89	6.08	5.81	6.0	6.06	6.17	5.98	6.2
A8-A9	T-shaped	6.27		6.29		6.28		6.32	
A10-C5	T-shaped	8.95		9.58		9.64		9.59	
C3-C4	Stacked	4.42		4.13		4.34		4.20	
C4-C5	Stacked	3.75	4.03	3.75	3.96	3.81	4.02	3.82	3.95
C8-C9	Stacked	4.07		4.08		4.15		4.00	
C9-C10	Stacked	3.88		3.86		3.78		3.78	
C1-C3	T-shaped	5.87		6.01		6.02		6.34	
C6-C8	T-shaped	5.87	5.87	5.92	5.97	6.04	6.03	5.82	6.08
C1-C2	Coplanar	6.19		6.14		6.29		6.11	
C1-C6	Coplanar	7.73	6.28	7.42	6.13	7.75	6.28	9.42	6.7
C6-C7	Coplanar	4.91		4.84		4.82		4.58	

Table S5: Difference in RMSF (ΔRMSF) values between the presence and absence of calcium ions for the residues THR494-THR501 and VAL565-ASN575 of MtrC in the reduced state. Δ RMSF = RMSF ($+Ca^{2+}$) - RMSF ($-Ca^{2+}$). The data were averaged over 3.3 μs equilibrium MD simulations.

Protein	Residues	Δ RMSF (Å)
MtrC	THR494	-2.11
MtrC	ALA495	-2.00
MtrC	CYS496	-1.75
MtrC	ALA497	-2.10
MtrC	ASN498	-2.51
MtrC	CYS499	-2.40
MtrC	HIS500	-1.83
MtrC	THR501	-2.27
MtrC	VAL565	-2.06
MtrC	GLU566	-2.34
MtrC	ASP567	-2.65
MtrC	ALA568	-3.10
MtrC	TYR569	-4.03
MtrC	GLY570	-3.66
MtrC	LEU571	-3.47
MtrC	ILE572	-2.89
MtrC	GLY573	-2.97
MtrC	GLY574	-2.09
MtrC	ASN575	-2.05

Table S6: Hydrogen bonds between the hemes (A10, C5, C1, and C6) and MtrCAB from 3.3 μs equilibrium MD simulations.

H-bonds between	Hydrogen bonds					
	Donor	Acceptor	Oxidized		Reduced	
			Occ. $(+Ca^{2+})$	Occ. $(-Ca^{2+})$	Occ. $(+Ca^{2+})$	Occ. $(-Ca^{2+})$
Heme and MtrCAB	ILE383-Main(MtrB)	A10-Side	36 %	6 %	35 %	2 %
	SER382-Side(MtrB)	A10-Side	31 %	5 %	52 %	2 %
	LYS389-Side(MtrB)	A10-Side	12 %	8 %	49 %	20 %
	SER284-Side(MtrA)	A10-Side	7 %	12 %	8 %	26 %
	SER477-Side(MtrB)	A10-Side	13 %	16 %	5 %	19 %
	LYS244-Side(MtrC)	C1- Side	30 %	33 %	62 %	75 %
	TRP239-Side(MtrC)	C1- Side	13 %	9 %	20 %	15 %
	VAL248-Main(MtrC)	C1- Side	11 %	7 %	35 %	12 %
	THR247-Main(MtrC)	C1- Side	4 %	3 %	27 %	11 %
	ASN251-Side(MtrC)	C6- Side	54 %	47 %	59 %	26 %
	LYS563-Side(MtrC)	C6- Side	24 %	16 %	17 %	38 %

Table S7: Non-bonded interactions between heme A10, along with its surrounding residues within 3.5 Å, and heme C5, along with its surrounding residues within 3.5 Å in the oxidized state. The data were averaged over 3.3 μs equilibrium MD simulations.

System	Non-bonded energy		
	Coulomb	vdW	Total
OM-MtrCAB-Ca(Ox)	-34.10	-14.41	-48.51 $\pm$ 5.3
OM-MtrCAB(Ox)	-18.42	-4.36	-22.78 $\pm$ 8.33
OM-MtrCAB-Ca(Red)	-15.1	-8.02	-23.12 $\pm$ 4.35
OM-MtrCAB(Red)	-18.50	-11.46	-29.96 $\pm$ 3.61

References

(S1) Edwards, M. J.; White, G. F.; Norman, M.; Tome-Fernandez, A.; Ainsworth, E.; Shi, L.; Fredrickson, J. K.; Zachara, J. M.; Butt, J. N.; Richardson, D. J.; Clarke, T. A. Redox linked flavin sites in extracellular decaheme proteins involved in microbe-mineral electron transfer. *Sci. Rep.* **2015**, *5*, 11677.

(S2) Jumper, J.; Evans, R.; Pritzel, A.; Green, T.; Figurnov, M.; Ronneberger, O.; Tunyasuvunakool, K.; Bates, R.; Žídek, A.; Potapenko, A.; Bridgland, A.; Meyer, C.; Kohl, S. A. A.; Ballard, A. J.; Cowie, A.; Romera-Paredes, B.; Nikolov, S.; Jain, R.; Adler, J.; Back, T.; Petersen, S.; Reiman, D.; Clancy, E.; Zielinski, M.; Steinegger, M.; Pacholska, M.; Berghammer, T.; Bodenstein, S.; Silver, D.; Vinyals, O.; Senior, A. W.; Kavukcuoglu, K.; Kohli, P.; Hassabis, D. Highly accurate protein structure prediction with AlphaFold. *Nature* **2021**, *596*, 583–589.

(S3) Firer-Sherwood, M. A.; Ando, N.; Drennan, C. L.; Elliott, S. J. Solution-based structural analysis of the decaheme cytochrome, MtrA, by small-angle X-ray scattering and analytical ultracentrifugation. *J. Phys. Chem. B* **2011**, *115*, 11208–11214.

(S4) Edwards, M. J.; White, G. F.; Butt, J. N.; Richardson, D. J.; Clarke, T. A. The crystal structure of a biological insulated transmembrane molecular wire. *Cell* **2020**, *181*, 665–673.

(S5) Schulz, G. E. The structure of bacterial outer membrane proteins. *Biochimica et Biophysica Acta (BBA)-Biomembranes* **2002**, *1565*, 308–317.

(S6) Breuer, M.; Rosso, K. M.; Blumberger, J. Flavin binding to the deca-heme cytochrome mtrc: insights from computational molecular simulation. *Biophys. J.* **2015**, *109*, 2614–2624.

(S7) Papadopoulos, J. S.; Agarwala, R. COBALT: constraint-based alignment tool for multiple protein sequences. *Bioinformatics* **2007**, *23*, 1073–1079.

(S8) Moser, C. C.; Dutton, P. L. Engineering protein structure for electron transfer function in photosynthetic reaction centers. *Biochim. Biophys. Acta, Bioenerg.* **1992**, *1101*, 171–176.
